# DWFAU-Net: A Clinically-Guided Dual-Window Fusion Attention U-Net for Coronary Artery Segmentation in CT Angiography

**DOI:** 10.3390/jimaging12100475

**Published:** 2026-10-01

**Authors:** Shafiya Qadeer Memon, Sania Bhatti, Muhammad Moazzam Jawaid, Gulzar Usman

**Affiliations:** 1 Department of Software Engineering, Mehran University of Engineering & Technology, Jamshoro 76062, Pakistan; shafiya.memon@faculty.muet.edu.pk (S.Q.M.); sania.bhatti@faculty.muet.edu.pk (S.B.); 2 School of Computer Science and Informatics, De Montfort University, Leicester LE1 9BH, UK; 3 Department of Community Medicine, Liaquat University of Medical and Health Sciences, Jamshoro 76090, Pakistan; gulzar@lumhs.edu.pk

**Keywords:** coronary artery segmentation, dual-window fusion attention U-Net, Hounsfield unit windowing, Frangi vesselness, clDice, Grad-CAM, MC-dropout uncertainty, patient-level cross-validation, explainable AI, cardiac CT angiography

## Abstract

Coronary artery segmentation in coronary computed tomography angiography (CCTA) is challenging because the vessels are small, low in contrast and anatomically variable. We propose a Dual-Window Fusion Attention U-Net (DWFAU-Net) in which two Hounsfield Unit windows, selected on validation patients, are processed by parallel encoders, fused by learnable 1 × 1 convolutions and decoded by a shared attention-gated decoder. Each window input combines the normalized CT image with a Frangi vesselness map and a Gaussian-smoothed image, and training uses a topology-aware BCE, Soft-Dice and clDice loss. On 16 Rotterdam volumes, under a patient-level protocol and without a region of interest, centerline or seed points, DWFAU-Net achieved a Dice Similarity Coefficient (DSC) of 0.691 and an IoU of 0.580 on unseen patients, with a stable DSC of 0.697 ± 0.012 over five trainings. In five-fold patient-level cross-validation, it achieved the highest slice DSC in folds 1–4 and the lowest mean 95th-percentile Hausdorff distance (30.55 pixels), with overall overlap statistically comparable to single-window and concatenation models. Ablations showed a consistent gain from the Frangi channel (volumetric Dice 0.712 vs. 0.684) and identified a clDice weight of 0.5 as the best of the tested weights, preserving patient-level overlap. The model was robust to ±50 HU contrast-enhancement shifts (DSC change ≤ 0.040) and segmented a patient in 0.31 s. A slice-level split gave a DSC of 0.870, showing how strongly the data-splitting protocol affects reported accuracy.

## 1. Introduction

Coronary artery disease (CAD) continues to be one of the major causes of death worldwide and cardiovascular diseases were responsible for an estimated 19.8 million deaths in 2022 [1]. This burden affects both developed and resource-limited health care systems alike [2]. Coronary computed tomography angiography (CCTA) has emerged as an important non-invasive imaging modality to assess the coronary arteries and identify atherosclerotic plaque in this setting. In many clinical settings, it is a useful non-invasive alternative to invasive catheter angiography [3]. Despite these benefits, automatic analysis of CCTA images remains a challenge. The coronary arteries are small, low-volume targets within a large 3D image, accounting for only a small fraction of the scan volume. They also vary greatly from one patient to another in their anatomy. Image quality is further affected by cardiac motion, partial-volume effects and noise. It becomes even more challenging to segment small distal vessels and especially branches with diameters of <1 mm, as well as to distinguish calcified, non-calcified and mixed plaque by subtle differences in image intensity and texture. These characteristics vary depending on image acquisition parameters and the patient anatomy [4,5,6,7]. Accurate automated segmentation and localization of coronary arteries can aid a number of downstream diagnostic tasks on CT, such as coronary stenosis analysis and coronary plaque burden analysis [8]. This is particularly important in the context of resource-scarce clinical environments where high-resolution and well-annotated medical imaging repositories may not be readily available. In such cases, data-dependent deep learning techniques may encounter challenges due to limited training data and lack of generalization performance across datasets. Moreover, manual interpretation of CCTA scans also demands significant clinical work and may result in inter-observer variation [9]. Automated methods for coronary artery segmentation are hence crucial for technical developments as well as for enhancing the efficiency, consistency and scalability of cardiovascular image analysis.

CNNs and encoder–decoder architectures such as U-Net [10] have made significant advances in medical image segmentation, but there are still significant challenges in coronary artery segmentation. Most current approaches treat the problem mainly as a data-driven prediction task and depend largely on information learned directly from image intensities. This makes it difficult to apply existing radiological knowledge and anatomical characteristics to the segmentation process. One important limitation concerns Hounsfield Unit (HU) windowing. Window level (WL) and window width (WW) are basic radiological parameters that have to be adjusted to control the visual contrast of the CT image. However, they are rarely studied systematically in coronary artery segmentation pipelines. Several of the current methods employ a single selected window without assessing if another clinically relevant HU window could provide additional vascular information [11,12]. Another drawback is that when there are several window configurations available, they are commonly used independently and not together. This could make it hard for the model to learn from the various representations’ contrast properties. In addition, the coronary arteries have a number of important structural characteristics such as their elongated tubular shape and connected tree-like structure, which are often assumed to be learned implicitly by the network. These are not always explicitly presented in the features fed to the model or promoted when optimizing [13,14,15]. Combined, these constraints show a gap between clinical image knowledge and purely data-driven segmentation. To be clinically useful, a coronary artery segmentation framework should leverage radiological contrast information better, take into account relevant vascular structure and connectivity, and offer information on model behavior and prediction uncertainty. This is especially relevant since small coronary branches may not be picked up if they are not segmented or segmented incorrectly and can lead to inaccurate downstream measurements, which could affect automated cardiovascular analysis.

To address these challenges, this study proposes a clinically guided Dual-Window Fusion Attention U-Net (DWFAU-Net) for coronary artery segmentation. The proposed framework considers two complementary HU window configurations, selected from literature-reported candidates using a grid-search proxy on validation patients. All the information from both windows is processed separately through two encoder paths and fused at different feature scales using learnable 1 × 1 convolutional fusion layers. A Frangi vesselness map and a Gaussian-smoothed version of the image are also added to the image representation for each HU-windowed image. This results in a three-channel input which integrates the original intensity data with vessel-specific morphological information and wider contextual information. The model thus also gets information about the local appearance, as well as the tubular properties of coronary vessels. Fused features are then decoded using an attention-guided U-Net decoder, based on the Attention U-Net architecture [16]. Within the skip connections, attention gates are applied to highlight relevant regions of the vasculature and to suppress responses from irrelevant background regions. To tackle the problem of segmentation overlap and structural continuity, the model is trained using a composite loss composed of Binary Cross-Entropy (BCE), Soft-Dice and topology-aware clDice [17]. While BCE and Soft-Dice optimize pixel-level classification and regional overlap, clDice is designed to encourage preservation of the connected centerline structure of the coronary vessel tree. Grad-CAM [18] and Monte-Carlo Dropout-based epistemic uncertainty estimation [19] are also included in the framework. While Grad-CAM gives information about the regions of the image that are important to the model’s predictions, Monte-Carlo Dropout gives information on the regions of the image the model is not confident in predicting. Together, these techniques provide evidence on model behavior beyond standard segmentation metrics.

The significance of the proposed work is that it aims to shift from a purely intensity-based segmentation strategy to a more clinically driven, structure-aware strategy. By combining radiologically motivated contrast selection with multi-window information, morphological feature enhancement, topology-aware optimization, attention-based refinement and uncertainty analysis, the proposed method aims to improve the accuracy, reliability and transparency required for clinically meaningful automated CCTA analysis. Because 2D slices from the same patient are highly correlated, all conclusions are based on patient-level evaluation; results under a slice-level split are also reported so that both protocols can be compared.

The objectives of this study are: (i) to select complementary HU windows for coronary segmentation in a reproducible way, using validation patients only; (ii) to develop a dual-window fusion Attention U-Net that combines these windows with vessel-oriented input features and a topology-aware loss; (iii) to evaluate the model at the patient level and compare it statistically with single-window and channel-concatenation designs under identical training; and (iv) to assess its reliability through centerline metrics, uncertainty calibration, robustness to contrast variation and computational cost. The key contributions of this work are as follows:

1. **Systematic HU Window Evaluation and Complementary Fusion:** Four candidate (WL, WW) configurations, three reported in the CCTA literature and one narrower empirical window, are evaluated using a grid-search proxy on validation patients, and the two best-ranked windows are used jointly. Rather than relying on a single configuration, the proposed framework leverages the complementary information present in both HU windows jointly.
2. **Morphology-Enhanced Multi-Channel Representation:** Three channels are used for each HU-windowed CCTA image: HU-normalized CT intensity, a Frangi vesselness map which emphasizes tubular vascular structures, and a Gaussian-smoothed image for context. This allows the model to learn appearance, morphology and local context at the same time.
3. **Dual-Window Fusion Attention U-Net:** DWFAU-Net is proposed, comprising two independent encoders that extract features from two different HU-window representations. The features at multiple scales are fused using learnable 1 × 1 convolutional fusion and then passed to the decoder.
4. **Attention-Based Spatial Refinement:** The skip connections include attention gates to enhance the attention to vessels and to diminish the effect of irrelevant background information. An ablation study compares this attention-gated design against an equivalent plain U-Net to examine the contribution of the attention mechanism.
5. **Topology-Aware Segmentation Optimization:** A composite BCE + Soft-Dice + clDice loss function is used to optimize pixel-level overlap and to encourage vascular connectivity. Its isolated effect and the sensitivity to the clDice weight are examined in ablation studies.
6. **Comprehensive Overlap and Boundary Evaluation:** IoU and Dice are used to assess overlap, and HD95 is used to measure boundary discrepancy while being less sensitive to extreme outliers than the maximum Hausdorff distance. Centerline metrics (skeleton recall, skeleton precision and overlap-until-first-error) are added to assess thin branches.
7. **Interpretability and Uncertainty Awareness:** To explore model attention and estimate epistemic uncertainty at the pixel level, Grad-CAM and Monte-Carlo Dropout are used, and the calibration of the uncertainty is quantified.
8. **Patient-Level, Statistically Tested Evaluation:** A patient-level protocol with validation-only model selection, five-fold patient-level cross-validation with paired statistical tests, and a comparison with single-window and two-channel concatenation designs trained under identical conditions are reported, together with slice-level results for comparison and contrast-shift, calcification and computational-efficiency analyses.

## 2. Literature Review

### 2.1. Coronary Artery Segmentation

Accurate identification and segmentation of coronary arteries in CT angiography are important for the diagnosis and treatment planning of patients with CAD. Traditionally, this task has been performed manually or with the support of semi-automated systems, making the process time-consuming and dependent on operator experience. Deep learning has provided approaches for automating coronary vessel analysis. For example, convolutional neural networks such as U-Net [10] can perform pixel-level segmentation of structures using relatively limited annotated data. Several studies have further developed this approach. A modified two-step U-Net was introduced to identify the location of blood vessels and then refine their boundaries to improve segmentation accuracy [13]. Recently, transformer-based methods have also been applied to medical image segmentation. Swin UNETR used self-supervised pre-training on large-scale 3D datasets to learn representations before fine-tuning [20]. Similarly, hybrid CNN and transformer-based architectures have been proposed for medical image segmentation [21]. Other approaches have combined U-Net-based networks with contextual transformer modules to improve the delineation of low-contrast vessels in CCTA images [22].

A continuing challenge in this area is the limited availability of large, accurately labeled medical imaging datasets. To reduce the dependence on extensive manual annotation, researchers have explored unsupervised and weakly supervised methods. For example, clustering has been used to segment coronary arteries without requiring fully annotated training data [23]. In another approach, a 3D CNN trained using basic or limited labels was applied to detect atherosclerosis directly from imaging data [24]. Software frameworks have also begun incorporating domain-adaptation modules alongside other learning paradigms to support model development across varying acquisition settings [25].

Data-efficient learning has become an important area of research in medical image analysis, with the goal of developing models that can perform well using relatively small amounts of labeled data. For coronary CTA segmentation, self-supervised pre-training has shown promising results, achieving a Dice score of up to 0.787 in a multi-institutional study [26]. However, its performance across other imaging protocols and clinical settings remains to be further investigated. In comparison, the CM-UNet model limited the loss in Dice to 15.2% when fine-tuned with only 18 labeled images instead of 500, although its evaluation was conducted on angiography images rather than volumetric CTA data [27]. Together, these studies suggest that reducing the need for labeled data is achievable, but maintaining strong performance across different datasets and imaging environments remains an important challenge.

### 2.2. Plaque Characterization and Risk Stratification

Beyond coronary lumen segmentation, deep learning has been applied to plaque characterization and cardiovascular risk stratification. Radiomics-based and deep learning approaches have been used to identify and classify calcified, non-calcified, and mixed plaques [4]. Specialized architectures such as PlaqueNet combine multi-scale features to segment plaques [7]. Multi-center studies have demonstrated that deep learning-derived plaque measurements can support cardiovascular risk prediction in clinical settings [28]. Other approaches combine CCTA findings with clinical variables to estimate cardiovascular outcomes [29]. Fine-tuning of pre-trained models has also been employed when labeled data are limited [30]. Additional research has explored plaque characterization using intravascular ultrasound and optical coherence tomography [31,32,33]. Some models jointly detect plaque and classify stenosis within a single network [34]. More recently, foundation models trained on large-scale CT datasets have been proposed to support a range of CT analysis tasks [35]. Collectively, these studies highlight the growing role of deep learning in plaque characterization and cardiovascular risk assessment.

### 2.3. Explainable AI in Cardiovascular Imaging

The use of Artificial Intelligence in clinical settings needs methods that support interpretation of model predictions. Grad-CAM [18] is among the most widely used techniques, generating heatmaps that highlight the image regions most influential to a model output. Several studies have shown that Grad-CAM derived attention regions align with areas radiologists consider diagnostically relevant, including applications in calcium detection [36] and coronary artery disease severity assessment [37,38]. Shapley Additive Explanations (SHAP) offers a complementary approach by quantifying the contribution of individual input features to model output, and has been applied to carotid artery analysis on CT angiography [39]. Beyond identifying influential image regions, quantifying predictive uncertainty is equally important for clinical trust. Bayesian approaches and Monte-Carlo Dropout [19] have been used to estimate the consistency of model prediction, supporting more reliable clinical decision-making [40,41]. The present framework builds on these approaches by incorporating both Grad-CAM and Monte-Carlo Dropout: Grad-CAM identifies the image regions most associated with the model’s prediction, while Monte-Carlo Dropout estimates the variability of those predictions, together providing complementary insight into model behavior for coronary artery segmentation.

## 3. Methodology

**Figure 1:**
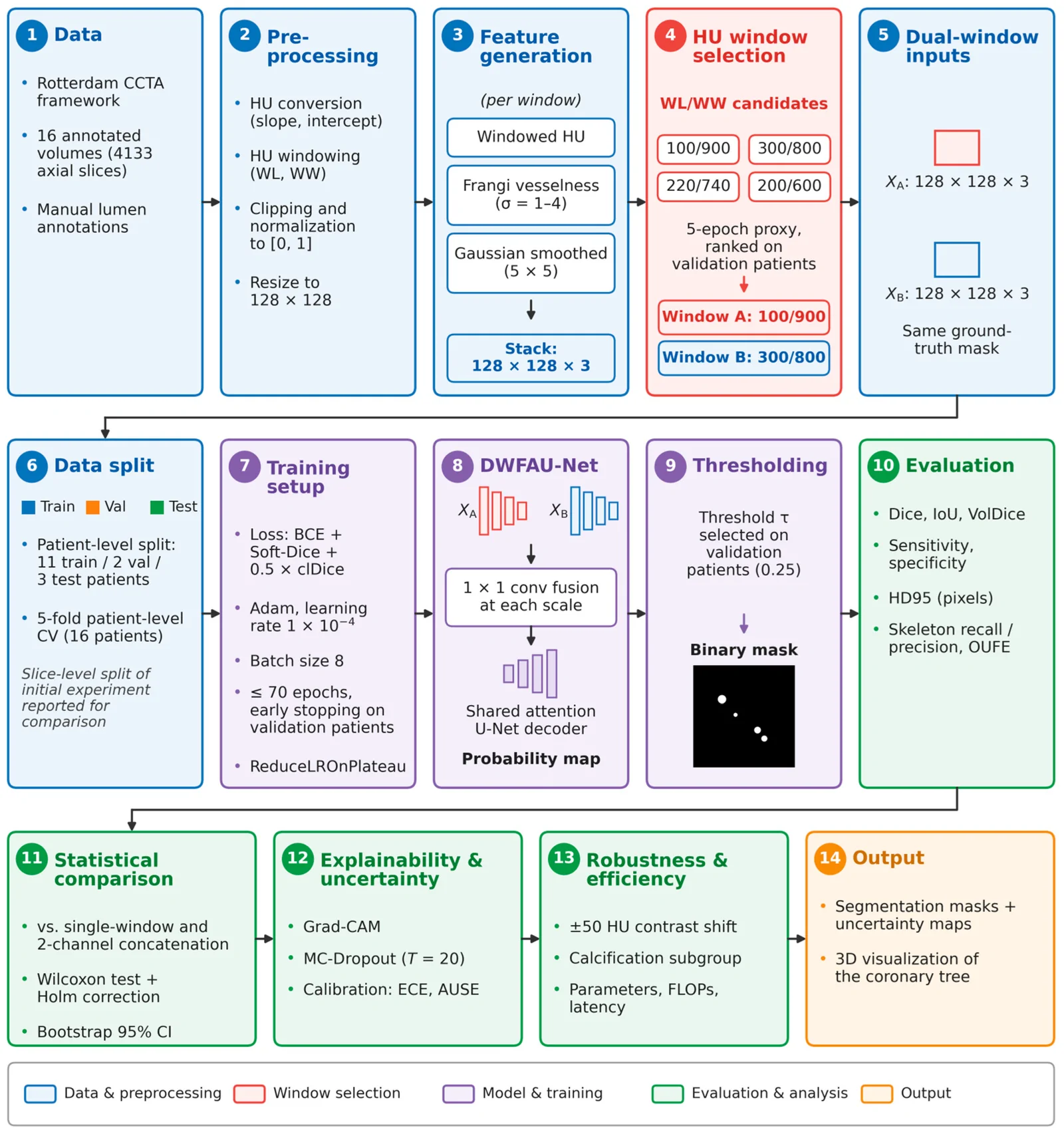
Overview of the proposed Dual-Window Fusion Attention U-Net pipeline. Stage 6 shows the patient-level data partitioning. The box colors indicate the stage groups given in the legend at the bottom of the figure.

This study proposes a Dual-Window Fusion Attention U-Net framework for automated coronary artery segmentation from cardiac CT angiography (CCTA) volumes, a systematic selection and fusion of literature-reported HU windowing parameters, and a topology-preserving training objective. The main steps of the pipeline, shown in Figure 1, are: (i) HU-window-based intensity normalization for the candidate windows; (ii) a grid-search ranking of the candidate windows on validation patients, retaining the two best-ranked windows; (iii) multi-channel feature construction combining raw CT intensity, Frangi vesselness response, and smoothed background context for each of the two windows; (iv) segmentation using a Dual-Window Fusion Attention U-Net architecture that fuses both windows through parallel encoders and a shared attention-gated decoder; (v) training with a composite Binary Cross-Entropy (BCE), Soft-Dice, and clDice topology-preserving loss; and (vi) quantitative evaluation, explainability analysis via Grad-CAM, and epistemic-uncertainty estimation via MC-Dropout. All data partitions are made at the patient level (Section 3.1), except for the component ablations in Section 4.4, which used a slice-level split (Section 3.7). An ablation study (input feature channels, network architecture, loss function, Frangi scale range, clDice weight and window fusion) was additionally conducted to isolate the contribution of each design choice.

### 3.1. Dataset and Preprocessing

This study employs the publicly available Rotterdam Coronary Artery Algorithm Evaluation Framework, a benchmark dataset developed for coronary artery lumen segmentation and stenosis quantification in CT angiography [42,43]. The framework comprises 48 CTA scans; its training set of 18 volumes, from which the data of this study were taken, was acquired with scanners from three vendors, with six volumes per vendor. Each case was accompanied by expert-annotated reference data represented as segment-wise three-dimensional discrete contours delineating the coronary artery lumen boundaries. The initial framework was devised primarily for the assessment of coronary lumens and quantification of stenosis, but reference data suitable for supervised coronary vessel segmentation were readily available. For two of the 18 volumes (volumes 16 and 17), no reference lumen masks were available after the reference contours were converted to voxel-wise segmentation masks; because supervised training and evaluation require a complete pixel-wise reference, these two volumes were excluded, and all experiments use the remaining 16 volumes (patients 00–15; 4133 axial slices). Additionally, the dataset contained scans obtained with different vendor equipment with variable image acquisition parameters, representing natural variations in scanner hardware and image acquisition characteristics. The data consisted of a number of CCTA exams obtained from various institutions from Siemens, Toshiba, and Philips scanners and reconstructed with clinically appropriate CTA protocols (Table 1). This diversity allowed for a heterogeneous environment for the test of the proposed approach.

To avoid data leakage between patients, all partitions were made at the patient (volume) level, so that every patient belongs to exactly one partition (the only exception is the slice-level component ablation described in Section 3.7). For the main experiment, the 16 patients were divided into 11 training, 2 validation and 3 test patients (random split, seed 42; validation patients 03 and 10; test patients 00, 01 and 05, 777 slices). The validation patients were used for all model-selection decisions (HU-window ranking, early stopping, learning-rate scheduling and decision threshold), and the test patients only for the reported results. In addition, five-fold patient-level cross-validation was performed over all 16 patients (Section 3.8).

Volumetric CT data and corresponding vessel masks were organized as paired DICOM slice series (“Volume” and “Mask” directories per case). Each DICOM slice was read using pydicom, and raw pixel intensities were converted to true Hounsfield Unit (HU) using the per-slice rescale slope and intercept stored in the DICOM header:

HU = pixel value × RescaleSlope + RescaleIntercept(1)

For each experiment, HU-based intensity windowing was subsequently applied to restrict the dynamic range according to window level (WL) and window width (WW). The lower and upper bounds were defined as:

lo = WL − WW/2(2)

hi = WL + WW/2(3)

The HU image was clipped to this interval and linearly normalized to [0, 1]

HU_clipped_ = clip(HU, lo, hi) (4)

HU_norm_ = (HU_clipped_ − lo)/(hi − lo) (5)

This preprocessing technique normalized intensities in the images and preserved the contrast characteristics of each HU window selected. Lastly, all of the axial CT images and masks were resampled to a fixed input size of 128 × 128 to aid computational efficiency of training and maintain uniformity. In general, the pre-processing pipeline ensured the same intensity scaling and spatial dimensions throughout the scans prior to feature extraction and model training.

### 3.2. HU Window Selection

**Figure 2:**
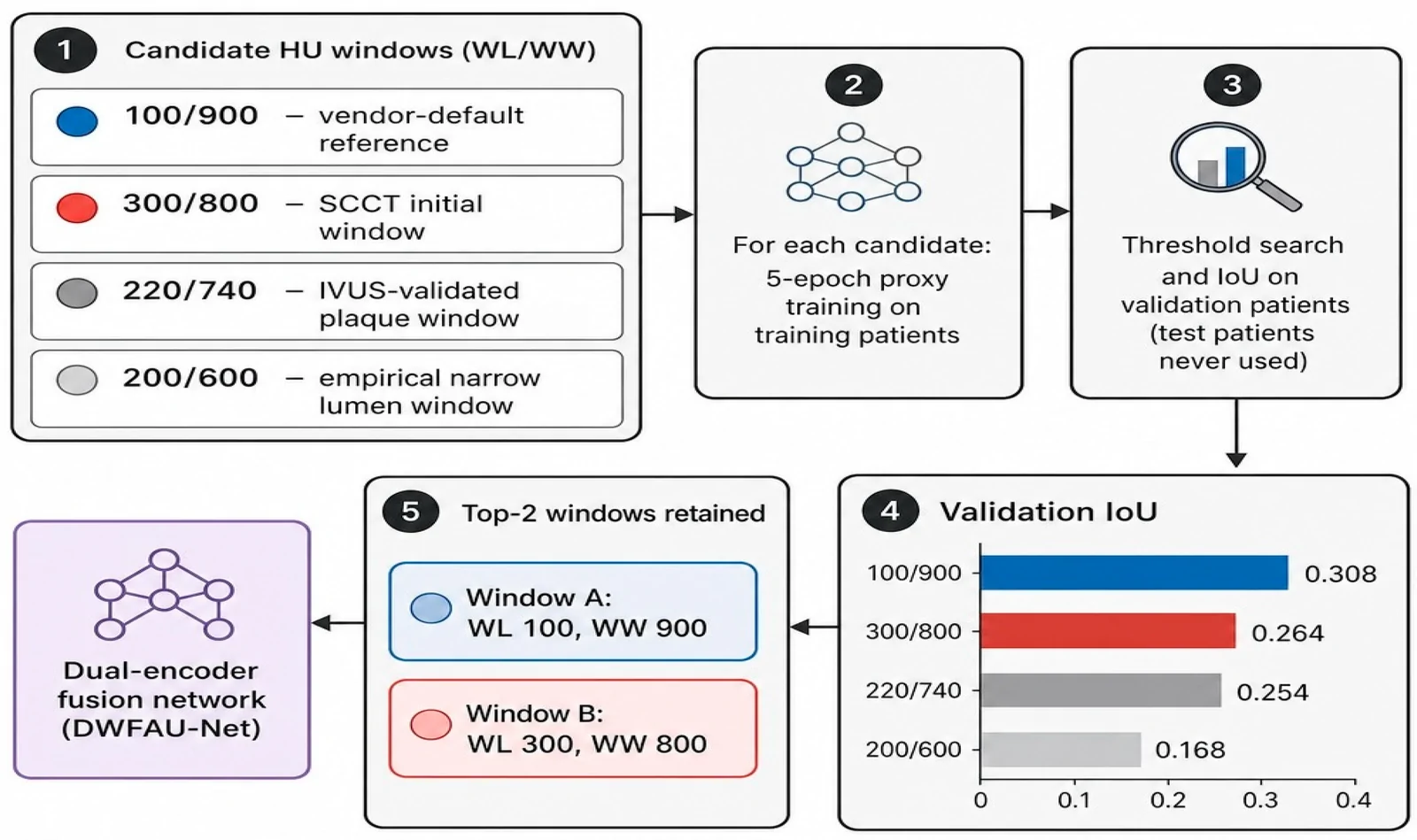
Workflow of HU Window Selection. The candidate windows are ranked on validation patients only. Sources of the candidate windows: 100/900, vendor-default reference window [12]; 300/800, initial window of the SCCT guidelines [11,44]; 220/740, IVUS-validated plaque-analysis window [45]; 200/600, empirical narrow lumen window.

This study employed two complementary Hounsfield Unit (HU) windows rather than a single, arbitrarily chosen setting. Both were selected from four candidate windows (Table 2): the initial window recommended by the SCCT guidelines [11,44], the vendor-default window used as the reference for conventional images by D’Angelo et al. [12], a fixed window validated against intravascular ultrasound for plaque volume analysis [45], and a narrower lumen-oriented window added empirically. No window parameter was derived from the intensity statistics of the study data. Each candidate was ranked by a 5-epoch proxy training evaluated on the validation patients only (Section 4.1), and the two best-ranked windows were retained as Window A (WL = 100, WW = 900) and Window B (WL = 300, WW = 800). Both were retained and jointly exploited within the Dual-Window Fusion architecture (Section 3.4), as each captures distinct, complementary contrast information. Figure 2 illustrates the window-selection workflow.

### 3.3. Multi-Channel Feature Construction

A three-channel representation of the input was developed for each of the two HU windows, to give information to the network other than the intensity of the original CT. The three channels were aimed at capturing complementary components of the image, such as the anatomical intensity information, the structures that resemble vessels, and the wider local image context.

*Channel 1: HU-Normalized CT Image.* The first channel contained the HU-windowed, normalized CT image. The anatomical intensity information was retained in this representation and was the basic image context provided to the network for segmenting.

*Channel 2: Frangi Vesselness Map.* The second channel was created by applying a Frangi vesselness filter, a multiscale Hessian-based method to enhance tubular structures [46]. The filter was applied to a sigma range of 1–4 pixels, which is the range expected for the width of the coronary lumen at the resized size of 128 × 128. The parameters were chosen as α = 0.5, β = 0.5, and γ = 15, and ‘black_ridges = False’ to enhance bright lumen structures in comparison with the darker surrounding areas. Each value in the resulting vesselness response was then scaled between 0 and 1 by dividing it by the maximum value in the map.

*Channel 3: Gaussian-Smoothed Image.* The third channel was a Gaussian-smoothed version of the input image with a Gaussian kernel size of 5 × 5. This channel kept low-frequency image information and a wider range of background context, which may assist the network in distinguishing between vascular structures and local noise, as well as other small intensity variations.

Each HU window then had the three representations stacked on top of each other to create a (128, 128, 3) input tensor. Each HU window was processed separately, resulting in two sets of three-channel input tensors being created. The following tensors were then fed to the two independent encoder branches of the proposed Dual-Window Fusion Attention U-Net (DWFAU-Net) as presented in Section 3.4.

### 3.4. Dual-Window Fusion Architecture

The proposed system does not rely only on the best-ranked HU window, because a single window may not be the optimal representation for every coronary structure. Different HU windows emphasize different contrasts between the enhanced lumen, the surrounding soft tissue and highly attenuating structures. Thus, the two best-ranked windows from the HU window selection process were used to create two representations of the same anatomical slice. These are considered to be Window A and Window B respectively. A 3-channel input for each window was created from the normalized CT image, the vesselness map of Frangi and the Gaussian-smoothed image. The obtained tensors were then separately sent to two encoder branches, both having the same network structure. Let *E*_A,*s*_ and *E*_B,*s*_ denote the encoder feature maps of windows A and B at scale *s*. At every encoder scale *s* ∈ {1, 2, 3, bottleneck}, the two encoders’ feature maps are fused via a learnable 1 × 1 convolution over their channel-wise concatenation:

*F_s_* = Conv_1×1_(Concat(*E*_A,*s*_, *E*_B,*s*_)) (6)

The fused feature maps {*F_s_*} are then passed to a single, shared decoder that mirrors the attention-gated design described in Section 3.5, producing one fused segmentation prediction per slice. This is an architectural fusion strategy distinct from simply concatenating both windows as six input channels into a single-encoder network since it allows each window-specific encoder to specialize before fusion, while the shared decoder and attention gates operate on a representation that already integrates complementary contrast information from both windows. This design is compared directly with a single-encoder model that receives both windowed images as input channels (Section 4.3).

### 3.5. Network Architecture

All encoder branches and shared decoder were based on the Attention U-Net architecture [16] that extended the conventional encoder–decoder U-Net architecture [10] with additive attention gates between the skip connections. The four convolutional stages of each encoder had 32, 64, 128 and 256 filters, respectively. Each stage consisted of two Conv2D–BatchNorm–ReLU layers; the first three stages were followed by 2 × 2 max-pooling to reduce the spatial dimensions, and the fourth stage (256 filters) formed the bottleneck. In the decoding process, an attention gate was used at each skip connection. Using coarse feature information extracted from the decoder as a gating signal (*g*) and the fine-resolution fused encoder features (*s*), the gate computed spatial attention coefficients in [0, 1]:

θ = Conv_1×1_(*s*) (7)

φ = Conv_1×1_(*g*) (8)

ψ = σ(Conv_1×1_(ReLU(θ + φ))) (9)

*s*′ = *s* ⊙ ψ (10)

**Figure 3:**
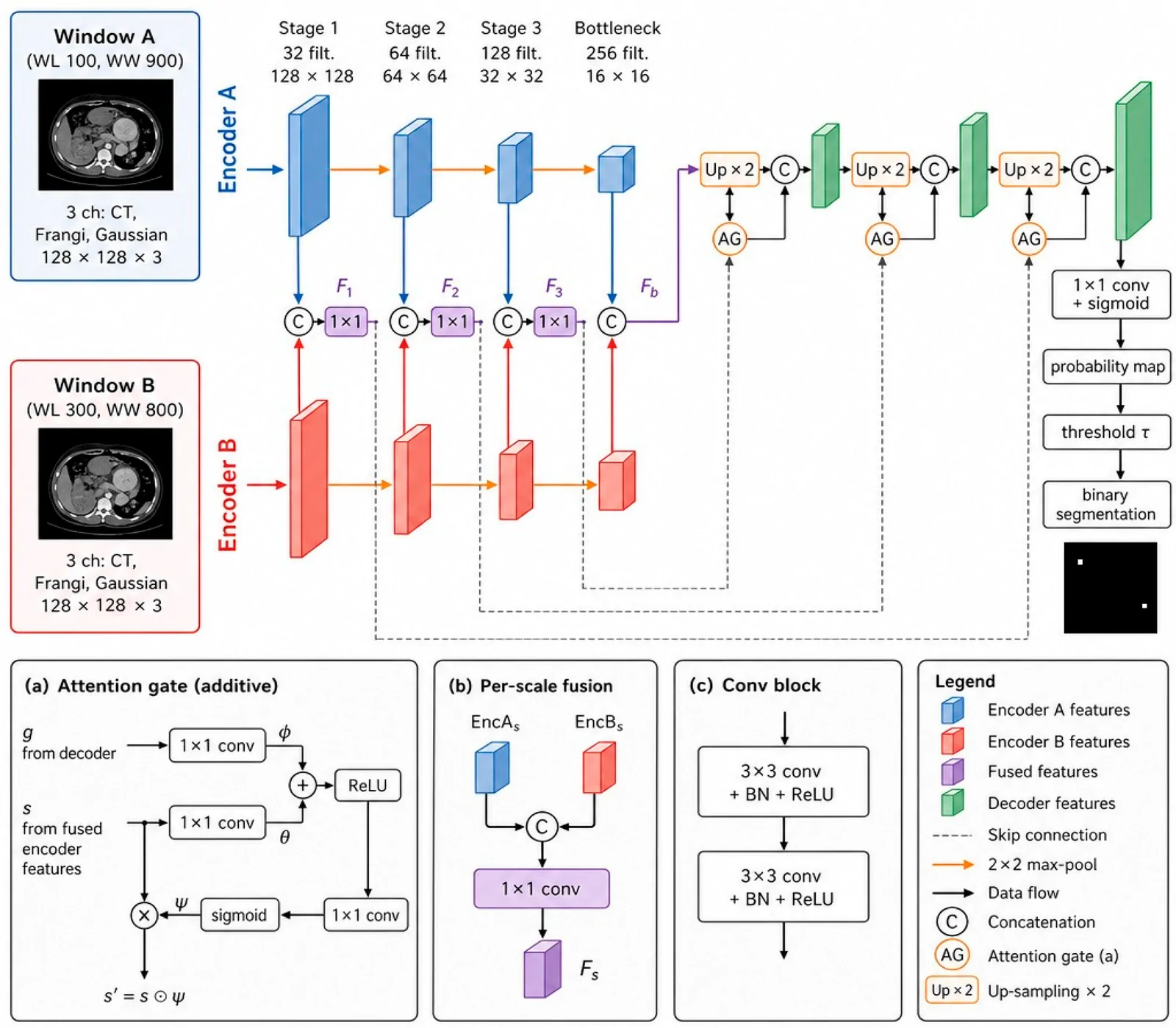
Proposed dual-window fusion Attention U-Net model architecture for coronary vessel segmentation. Window labels show the windows selected in this study.

The resulting coefficients were used to regulate the information flowing through each skip connection. The decoder features were upsampled and concatenated with the attention-weighted skip features, in which responses from less relevant background regions were reduced. This enabled the network to focus more on the small and potentially low-contrast coronary vessels and suppress the effects of irrelevant activations. In addition, as an architectural ablation baseline, a plain U-Net was also implemented to explore the specific effect of the attention mechanism. This comparison model had the same number of filters and the same encoder/decoder depth, without attention gates. Dropout layers (rate 0.2) were placed after the bottleneck and after the first two decoder blocks; they are inactive during standard inference and are used only for the MC-Dropout uncertainty analysis (Section 3.9). The network is two-dimensional and is applied to each axial slice independently. The architecture is shown in Figure 3.

Algorithm 1 summarizes the procedure of the proposed method.
**Algorithm 1:** Proposed Dual-Window Topology-Preserving Fusion Framework**Input**: CCTA DICOM Image *I*, Corresponding ground-truth mask *Y* **Output**: Final Coronary artery segmentation *S* **Start**: 1. Load *I* and *Y* 2. Apply two complementary HU Windows:*W*_A_ = HU window with highest validation performance *W*_B_ = second-best complementary HU window3. For each slice of *I* generate:*I*_A_ = Window(*I*, *W*_A_) *I*_B_ = Window(*I*, *W*_B_)4. Construct Multi-feature representation for each window:*F*_A_ = [*I*_A_, Frangi(*I*_A_), GaussianBlur(*I*_A_)] *F*_B_ = [*I*_B_, Frangi(*I*_B_), GaussianBlur(*I*_B_)]5. Extract hierarchical features independently:{*E*_A,1_, *E*_A,2_, *E*_A,3_, *E*_A,b_} = Encoder_A_(*F*_A_) {*E*_B,1_, *E*_B,2_, *E*_B,3_, *E*_B,b_} = Encoder_B_(*F*_B_)6. Perform learnable multi-scale feature fusion:*F*_1_ = Conv_1×1_([*E*_A,1_ ‖ *E*_B,1_]) *F*_2_ = Conv_1×1_([*E*_A,2_ ‖ *E*_B,2_]) *F*_3_ = Conv_1×1_([*E*_A,3_ ‖ *E*_B,3_]) *F*_b_ = Conv_1×1_([*E*_A,b_ ‖ *E*_B,b_])7. Apply shared attention-guided decoder:*D*_1_ = Decoder(*F*_b_, Attention(*F*_3_)) *D*_2_ = Decoder(*D*_1_, Attention(*F*_2_)) *D*_3_ = Decoder(*D*_2_, Attention(*F*_1_))8. Generate Probability Segmentation Map:*P* = Sigmoid(Conv_1×1_(*D*_3_))9. Optimize Network using Topology-aware Hybrid Loss:*L*_total_ = *L*_BCE_ + *L*_Dice_ + λ *L*_clDice_10. Train network using training parameters 11. Obtain final binary segmentation:*S* = 1 if *P* ≥ τ, otherwise 012. Return *S*

### 3.6. Loss Function

The network was trained using a composite loss combining BCE, Soft-Dice, and a topology-preserving clDice term [17]:

*L* = *L*_BCE_(*y*, *ŷ*) + *L*_Dice_(*y*, *ŷ*) + λ · *L*_clDice_(*y*, *ŷ*) (11)

*L*_Dice_ = 1 − (2 · Σ(*y* · *ŷ*) + ε)/(Σ*y* + Σ*ŷ* + ε), λ = 0.5 (12)

The Soft-Dice term directly optimizes for regional overlap and is robust to the severe foreground–background class imbalance inherent to thin vessel structures, while the BCE term provides a stable pixel-wise gradient signal. The clDice term [17] measures agreement between each mask’s differentiable morphological skeleton (soft-skeletonized via iterative soft-erosion/soft-dilation) and the other mask’s segmentation, explicitly penalizing breaks in the predicted vessel centerline that pixel-overlap losses alone would not detect, a property directly relevant to a tubular structure such as a coronary artery, where a fragmented centerline is a clinically meaningful error even when pixel-wise overlap remains high. For comparison, BCE only, Focal + Dice [47], the BCE + Soft-Dice combination without the clDice term, and BCE + Soft-Dice + clDice with weights of 0.25, 0.5 and 1.0 were evaluated in the ablation study (Section 4.4).

### 3.7. Training Protocol and Ablation Design

Table 3 shows the experimental setup during training of the proposed model. All models compared under the patient-level protocol (the proposed model and the single-window and two-channel baselines) were trained with the same recipe: at most 70 epochs, early stopping on the validation loss, ReduceLROnPlateau, on-the-fly augmentation (horizontal flip, small rotation and translation) and three-fold oversampling of slices containing vessel. For each model, the decision threshold was selected from 0.20–0.70 by maximizing the mean Dice on the validation patients; post-processing was not applied.

To isolate the contribution of key design decisions, an ablation study was conducted (input feature channels, network architecture, loss function, Frangi scale range, clDice weight and window fusion). Each variant was trained with the same recipe (a maximum of 70 epochs with early stopping); early stopping was not triggered in any variant, so all variants were trained for exactly 70 epochs and therefore had an identical training budget. These ablations were run under a slice-level split, in which early stopping monitored the held-out slices; their results are therefore interpreted only as a comparison between variants (Section 4.4). In addition, the clDice term and the Frangi channel were ablated under the patient-level protocol with the same recipe as the final model (Section 4.4).

### 3.8. Evaluation Metrics and Statistical Analysis

Overlap was measured with the Dice coefficient and IoU, computed per slice and averaged, and with the volumetric Dice (VolDice), computed over all slices of a patient. Sensitivity, specificity and the 95th-percentile Hausdorff distance (HD95) were also computed. Because the DICOM files available to us do not contain the in-plane pixel spacing, HD95 is reported in pixels of the 128 × 128 grid and is used only for comparisons within this study. Centerline agreement was measured on the skeletons of the reference and predicted masks with skeleton recall, skeleton precision and overlap-until-first-error.

In five-fold patient-level cross-validation (16 patients; two training patients per fold used for validation), DWFAU-Net was compared with (i) a single-window Attention U-Net using the best-ranked window with the same three input channels (CT, Frangi and Gaussian) and (ii) a single-encoder Attention U-Net receiving only the two windowed CT images as two input channels, all trained with the same recipe. The two HU windows were those ranked on the validation patients of the main split and were kept fixed in all folds; because two of these patients are test patients in folds 4 and 5, window selection was not repeated inside each fold. Per-patient results were compared with paired two-sided Wilcoxon signed-rank tests [48], with Holm correction [49] applied per metric over the two comparisons, and 95% confidence intervals of the mean paired difference were obtained by bootstrapping (10,000 resamples).

### 3.9. Explainability and Uncertainty Analysis

To assess the interpretability and clinical trustworthiness of the model’s predictions, Grad-CAM [18] was applied to the final convolutional layer of the trained dual-input network. Class-activation gradients were backpropagated with respect to the mean predicted output, spatially averaged to obtain channel weights, and upsampled to the input resolution to produce a heatmap highlighting image regions most influential to the segmentation decision. Heatmaps were overlaid on the original CT slices to qualitatively examine whether the network’s attention is located near the coronary vessels rather than on background structures. In addition, epistemic uncertainty was estimated via Monte-Carlo Dropout [19]: the dropout layers (rate 0.2) after the bottleneck and the first two decoder blocks were kept active at inference time, and *T* = 20 stochastic forward passes were drawn per test slice (the batch-normalization layers were also in training mode during these passes). The pixel-wise mean and standard deviation of the *T* predictions were used to generate the mean prediction and the per-slice epistemic-uncertainty map; the MC-Dropout passes were used only for this uncertainty analysis, and all reported segmentation metrics were computed from a single deterministic forward pass. Calibration was quantified, following [40], with the expected calibration error (ECE), the area under the sparsification error curve (AUSE), and the correlation between per-slice mean uncertainty and segmentation error (1 − Dice). Algorithm 2 depicts the mathematical procedure for Grad-CAM and MC-Dropout-based epistemic uncertainty estimation in the proposed dual-window fusion attention U-Net.
**Algorithm 2:** Explainability and Uncertainty Analysis**Input:** – Trained dual-window fusion model *f*_θ_ – Dual-Window Input *X*_A_, *X*_B_ – Target Convolutional Feature Maps *A* = {*A*^1^, …, *A^K^*} – Number of stochastic passes *T* = 20 – Decision Threshold τ (selected on the validation patients) **Output:** – Grad-CAM Heatmap *H*_CAM_ – Predictive mean μ – Epistemic Uncertainty Map *Z* – Binary Segmentation *Ŷ* (for the uncertainty analysis only; reported metrics use a single deterministic pass) **Begin:** 1. *P* ← *f*_θ_(*X*_A_, *X*_B_) Compute scalar segmentation response:*s* ← (1/(*H* × *W*)) Σ*_i_*_,*j*_ *P_ij_*2. For *k* = 1 to *K* do Compute Grad-CAM Weight:3. α*_k_* ← (1/(*H* × *W*)) Σ*_i_*_,*j*_ ∂*s*/∂*A^k^_ij_*End For 4. Generate *H*_CAM_5. *H*_CAM_ ← ReLU(Σ*_k_* α*_k_ A^k^*)Resize Grad-CAM Map6. *H*_CAM_ ← Resize(*H*_CAM_, *H*, *W*)Normalize Grad-CAM7. *H*_CAM_ ← (*H*_CAM_ − min(*H*_CAM_))/(max(*H*_CAM_) − min(*H*_CAM_))Enable Dropout layers during inference 8. For *t* = 1 to *T* do Generate Stochastic Prediction9. *P*^(*t*)^ ← *f*_θ_^(*t*)^(*X*_A_, *X*_B_)End for 10. Compute μ11. μ*_ij_* ← (1/*T*) Σ*_t_ P*^(*t*)^*_ij_*Compute *Z*12. *Z_ij_* ← √[(1/*T*) Σ*_t_* (*P*^(*t*)^*_ij_* − μ*_ij_*)^2^]13. Generate *Ŷ*14. *Ŷ_ij_* ← 1 if μ*_ij_* ≥ τ, otherwise 015. Return *H*_CAM_, μ, *Ŷ*, *Z*

## 4. Results

### 4.1. HU Window Selection Results

The results of the window selection procedure described in Section 3.2 are summarized in Table 4. A proxy training experiment with 5 epochs was performed for each candidate window, followed by optimization of the candidate window thresholds to find the threshold which maximized the IoU on the validation patients. The conventional window (WL = 100, WW = 900) had the best performance with a validation IoU of 0.308 at a threshold of 0.45, followed by the standard CCTA review (SCCT) window (WL = 300, WW = 800) with 0.264 at a threshold of 0.30. These two windows were assigned to Window A and Window B, respectively. The proxy thresholds were only used to rank the candidate windows; the decision threshold of the fully trained fusion model was selected separately on the validation patients (Section 4.2).

### 4.2. Final Model Performance

After ranking the HU windows, the proposed Dual-Window Fusion Attention U-Net was trained for a maximum of 70 epochs using the combined BCE, Soft-Dice, and clDice loss. Early stopping and learning-rate scheduling were applied during training to support stable convergence, both monitored on the validation patients. For final testing, segmentation outputs were converted to binary masks using a threshold of 0.25 selected on the validation patients. The resulting performance on the three held-out test patients (777 slices) is presented in Table 5, together with the result of our initial slice-level experiment.

The final model achieved an IoU of 0.580 and a Dice coefficient of 0.691, showing agreement between the predicted and reference coronary vessel masks. A sensitivity of 0.697 shows that about seven in ten vessel pixels were correctly identified. The specificity of 0.9997 shows the model’s ability to distinguish vessel regions from the predominantly larger background area, which is expected in thin-vessel segmentation tasks. The mean HD95 was 22.20 pixels at the 128 × 128 image resolution. However, the high standard deviation (±22.79) shows that boundary errors varied considerably across individual test slices. Larger deviations may happen in challenging regions, such as vessel bifurcations, thin distal branches or slices containing only partially visible vessel structures. The skeleton recall of 0.697 indicates that about 70% of the reference centerline was covered by the prediction.

To quantify the effect of the data-splitting protocol, the same dual-window fusion architecture was also trained and tested with a conventional slice-level split, in which the 2D slices of all patients are pooled before they are randomly assigned to the training and test sets. This split yielded a Dice of 0.870 and an IoU of 0.783 (Table 5). In that setting, adjacent slices from the same patient, which are anatomically very similar, are present in both the training and the test set, so the network has already seen the test patients during training. Under the patient-level protocol, in which every patient belongs to only one partition and all model selection is done on separate validation patients, the Dice was 0.691. The slice-level experiment used a fixed threshold of 0.5 instead of a threshold selected on validation patients, so the difference of about 0.179 mainly reflects the splitting protocol, together with this configuration difference. Both results are reported for transparency, but only the patient-level values are used as the estimate of performance on unseen patients and for the conclusions of this study.

### 4.3. Five-Fold Patient-Level Cross-Validation

**Figure 4:**
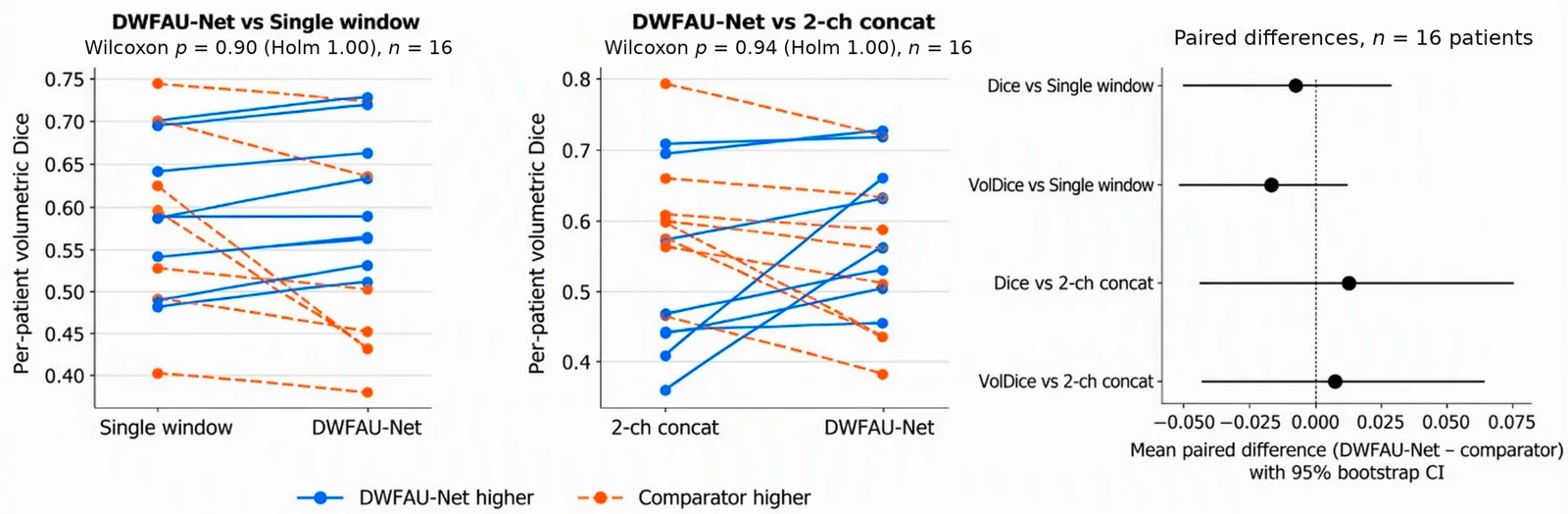
Per-patient volumetric Dice of DWFAU-Net versus the single-window (**left**) and two-channel concatenation (**middle**) models in five-fold patient-level cross-validation, and mean paired differences with 95% bootstrap confidence intervals (**right**).

To check that the result is stable with the limited data, the evaluation was extended to five-fold patient-level cross-validation, in which each of the 16 patients was tested exactly once. DWFAU-Net was compared with a single-window model and a two-channel concatenation model trained under identical settings (Table 6, Table 7 and Table 8, Figure 4).

The three models achieved similar overlap, and none of the paired differences was statistically significant. The 95% confidence intervals of the paired differences in volumetric Dice were narrow (−0.054 to 0.011 versus the single-window model and −0.044 to 0.063 versus the concatenation model; Table 7), which indicates that large differences between the designs are unlikely on this dataset. DWFAU-Net had the lowest mean HD95 (30.55 px; lower than the single-window model in 10 of 16 patients). In each of folds 1–4, it achieved the highest slice Dice of the three models (0.656, 0.455, 0.576 and 0.479, compared with 0.638, 0.393, 0.558 and 0.475 for the single-window model and 0.625, 0.429, 0.532 and 0.387 for the concatenation model), and its volumetric Dice was equal to or higher than that of the single-window model in folds 2–4 (Table 8); over the 13 patients of folds 1–4, its mean slice Dice was 0.025 higher and its mean volumetric Dice 0.008 higher than those of the single-window model. In fold 5 (patients 03, 06 and 12), however, its performance dropped markedly (slice Dice 0.257 versus 0.405, volumetric Dice 0.439 versus 0.568), and this single fold accounts entirely for the slightly lower mean overlap of DWFAU-Net over all 16 patients (Table 6). Repeating fold 5 with a second training seed for all three models gave the same pattern (VolDice 0.465 for DWFAU-Net, 0.541 for the single-window and 0.471 for the concatenation model), and per-patient VolDice changed by up to 0.163 between the two seeds. Per-patient VolDice of DWFAU-Net ranged from 0.380 to 0.728 and was not correlated with the mean lumen attenuation of the patients (Spearman ρ = 0.224, *p* = 0.405). Fold 5 was the weakest fold of DWFAU-Net (volumetric Dice 0.439, compared with 0.653 in fold 1), but not of the other two models, whose lowest volumetric Dice occurred in fold 2 (single window, 0.530) and fold 4 (concatenation, 0.492) (Table 8). In the repeated run of this fold, for which per-patient values were recorded, most of the drop came from patient 03, which was difficult for all three models (volumetric Dice 0.31–0.45, HD95 56–58 px) and has the second-lowest mean lumen attenuation of the cohort (176.5 HU; cohort range 173.9–457.0 HU). In the same run, the decision threshold selected on the two validation patients of the fold was 0.60 for DWFAU-Net and 0.70 for the concatenation model, but 0.30 for the single-window model; the two high-threshold models under-segmented the test patients (sensitivity 0.16–0.38 for DWFAU-Net versus 0.39–0.68 for the single-window model), and the single-window model achieved the highest volumetric Dice. The weaker fold-5 result of DWFAU-Net is therefore largely explained by the threshold selected on only two validation patients, which reflects the general variability of validation-based model selection in this setting: in the patient-level ablation, the threshold selected for the full model ranged from 0.20 to 0.55 across five seeds, and the number of trained epochs from 24 to 63. In cross-validation, DWFAU-Net, which has 1.68× the parameters of the single-encoder model, also stopped earlier on average than the single-window model (39.6 ± 9.2 versus 52.4 ± 4.0 epochs), which may make it more sensitive to this variability.

### 4.4. Ablation Study

**Figure 5:**
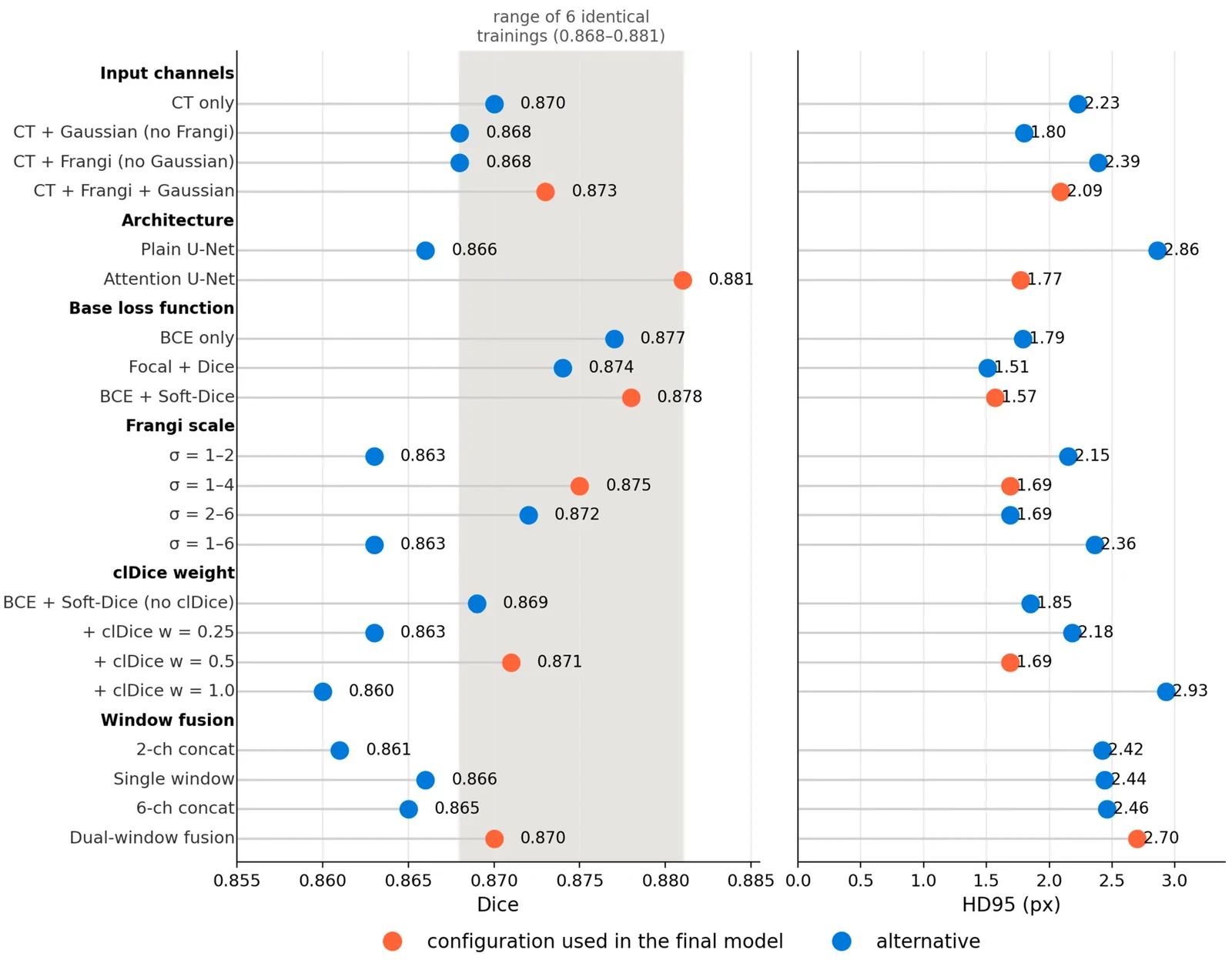
Ablation results (Dice and HD95) under the slice-level protocol. Orange points mark the configuration used in the final model; the grey band shows the range of Dice over six trainings of one identical configuration.

Table 9 and Figure 5 summarize the ablation results. All variants were trained with the same recipe for 70 epochs under a slice-level split; the values are therefore optimistic and are used only to compare variants.

For the input channels, the full three-channel input (CT + Frangi + Gaussian) gave the highest IoU (0.794) and Dice (0.873), compared with 0.870 for CT only and 0.868 without the Frangi channel. For the architecture, the Attention U-Net achieved a Dice of up to 0.881 compared with 0.866 for the plain U-Net. For the base loss function, BCE + Soft-Dice, which is used in the final model, gave the highest IoU (0.798) and Dice (0.878), compared with 0.877 for BCE only and 0.874 for Focal + Dice, while Focal + Dice gave the lowest HD95 (1.51 px). The clDice weight had a clear effect: *w* = 0.5 gave the best Dice (0.871) and HD95 (1.69 px) among the clDice variants, whereas *w* = 1.0 gave the lowest Dice (0.860) and the highest HD95 (2.93 px) of all variants in Table 9, and *w* = 0.25 also reduced the Dice (0.863); *w* = 0.5 was therefore used in the final model. Compared with the loss without clDice (Dice 0.869, HD95 1.85 px), *w* = 0.5 improved the Dice by 0.002 and the HD95 by 0.16 px. The same configuration trained six times gave Dice values between 0.868 and 0.881. This run-to-run variation is as large as the differences between the variants, so the individual contributions of the Frangi channel, the attention gates, the base loss function and the clDice term could not be isolated reliably at this sample size. These components are kept in the final model as design choices suited to tubular structures; the effects of the Frangi channel and the clDice term were therefore also examined with a patient-level ablation (Table 10). For window fusion, DWFAU-Net gave a Dice of 0.870, compared with 0.866 for the single window and 0.861 and 0.865 for the 2- and 6-channel concatenation models; these differences are also within run-to-run variation, in agreement with the patient-level cross-validation (Section 4.3).

For the patient-level ablation, three variants of DWFAU-Net were trained on the main split (11 training, 2 validation and 3 test patients) with identical settings, each changing only one component: the full model (P0), the model without the clDice term (P1) and the model without the Frangi channel (P2). Each variant was trained with the same five seeds (42, 7, 1, 2 and 3). Early stopping, the learning-rate schedule and the decision threshold were selected on the validation patients, and the three held-out test patients (777 slices) were used for evaluation (Table 10).

Removing the Frangi channel lowered the mean Dice from 0.697 to 0.677, the volumetric Dice from 0.712 to 0.684 and the IoU from 0.561 to 0.539. The full model had the higher Dice in four of the five seeds and the higher mean volumetric Dice in all three test patients (0.738 versus 0.701, 0.725 versus 0.710 and 0.674 versus 0.641), and its results varied less between seeds (standard deviation of the Dice 0.012 versus 0.028). The Wilcoxon signed-rank test gave *p* = 0.31 for the Dice and *p* = 0.13 for the volumetric Dice; with five paired trainings, the smallest two-sided *p*-value this test can reach is 0.0625, so significance at the 0.05 level was not attainable in this design, but the direction of the effect was consistent across seeds and patients. Skeleton recall and overlap until first error were unchanged; skeleton precision was slightly higher with the Frangi channel (0.705 versus 0.674), within the seed-to-seed spread. With the clDice term at *w* = 0.5, the overlap of the model was maintained: the mean Dice (0.696 versus 0.697), volumetric Dice (0.711 versus 0.712), skeleton recall (0.714 versus 0.706) and overlap until first error (0.435 versus 0.440) were almost identical, and the full model was better in two of the five seeds. The five trainings of the full model gave Dice values between 0.678 and 0.708, a range that includes the 0.691 of the main model (mean 0.697), which indicates that the main result reported in Section 4.2 is representative of repeated trainings. In summary, the patient-level ablation supports a consistent contribution of the Frangi channel to volumetric overlap (an increase of 0.028, about 4%) and to stability between trainings, whereas at *w* = 0.5 the clDice term changed only the skeleton precision slightly (0.705 versus 0.690).

### 4.5. Training Dynamics

**Figure 6:**
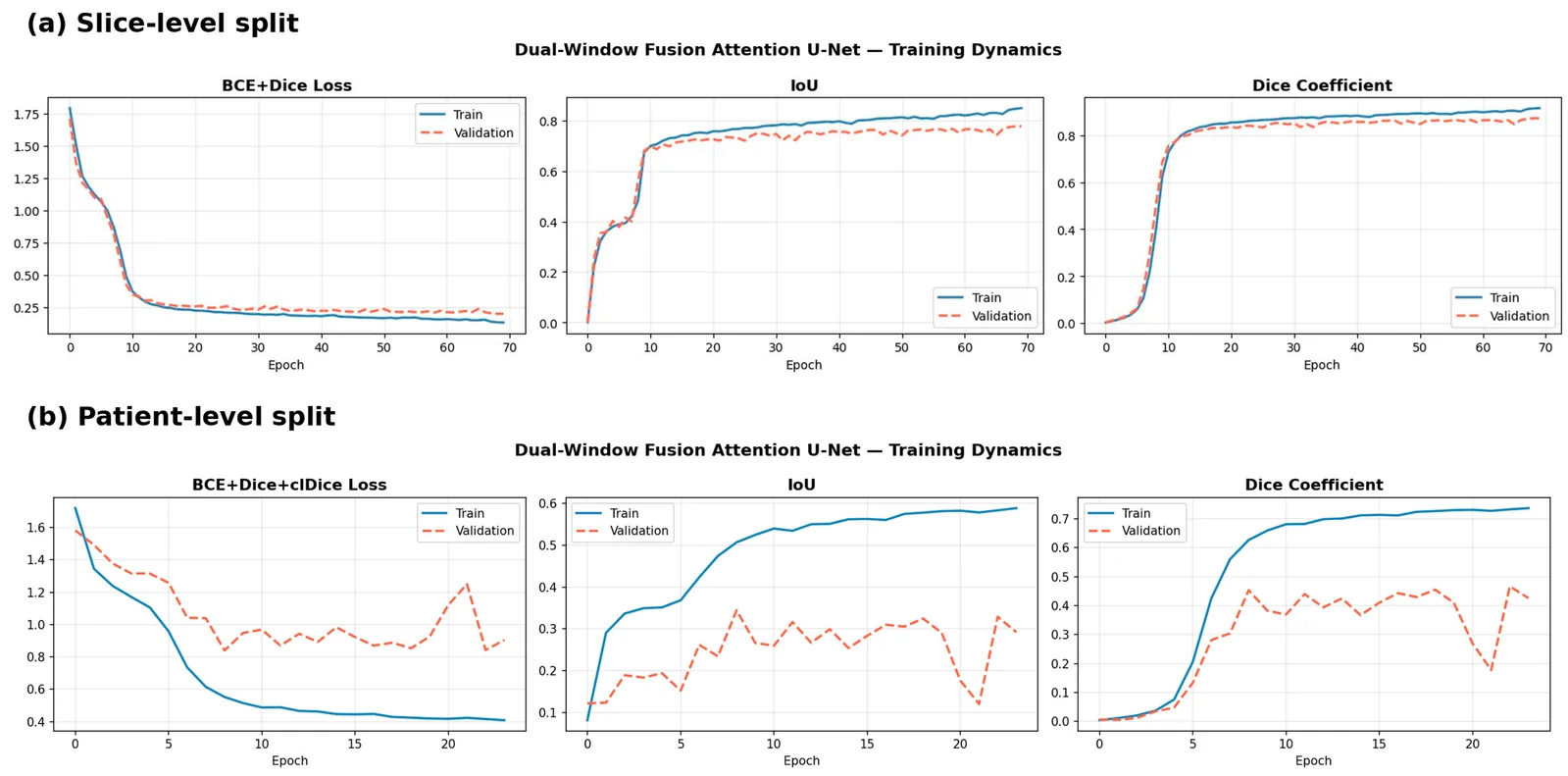
Training and validation curves (loss, IoU and Dice coefficient). (**a**) Slice-level split (single training run); (**b**) proposed dual-window fusion model with BCE + Soft-Dice + clDice loss under the patient-level split, with early stopping on validation patients.

Figure 6 presents the training and validation curves for (a) the model trained under the slice-level split and (b) the proposed fusion model under the patient-level split. In the slice-level experiment (Figure 6a), during the initial epochs, both training and validation loss decreased rapidly, while the IoU and Dice coefficient showed a corresponding improvement. As training progressed, the validation metrics gradually stabilized, whereas the training metrics continued to improve. The validation curves followed the training curves closely, because the validation slices came from the same patients as the training slices. Under the patient-level split (Figure 6b), the validation metrics stabilized after about ten epochs and then fluctuated, with a clear gap between training and validation performance. This gap reflects the difference between training patients and unseen validation patients, and it shows visually why the slice-level results were optimistic. The learning-rate scheduling and early-stopping strategies described in Section 3.7 were used to manage the training process by reducing the learning rate when validation improvement slowed and restoring the model weights associated with the best validation performance.

### 4.6. Qualitative Results

**Figure 7:**
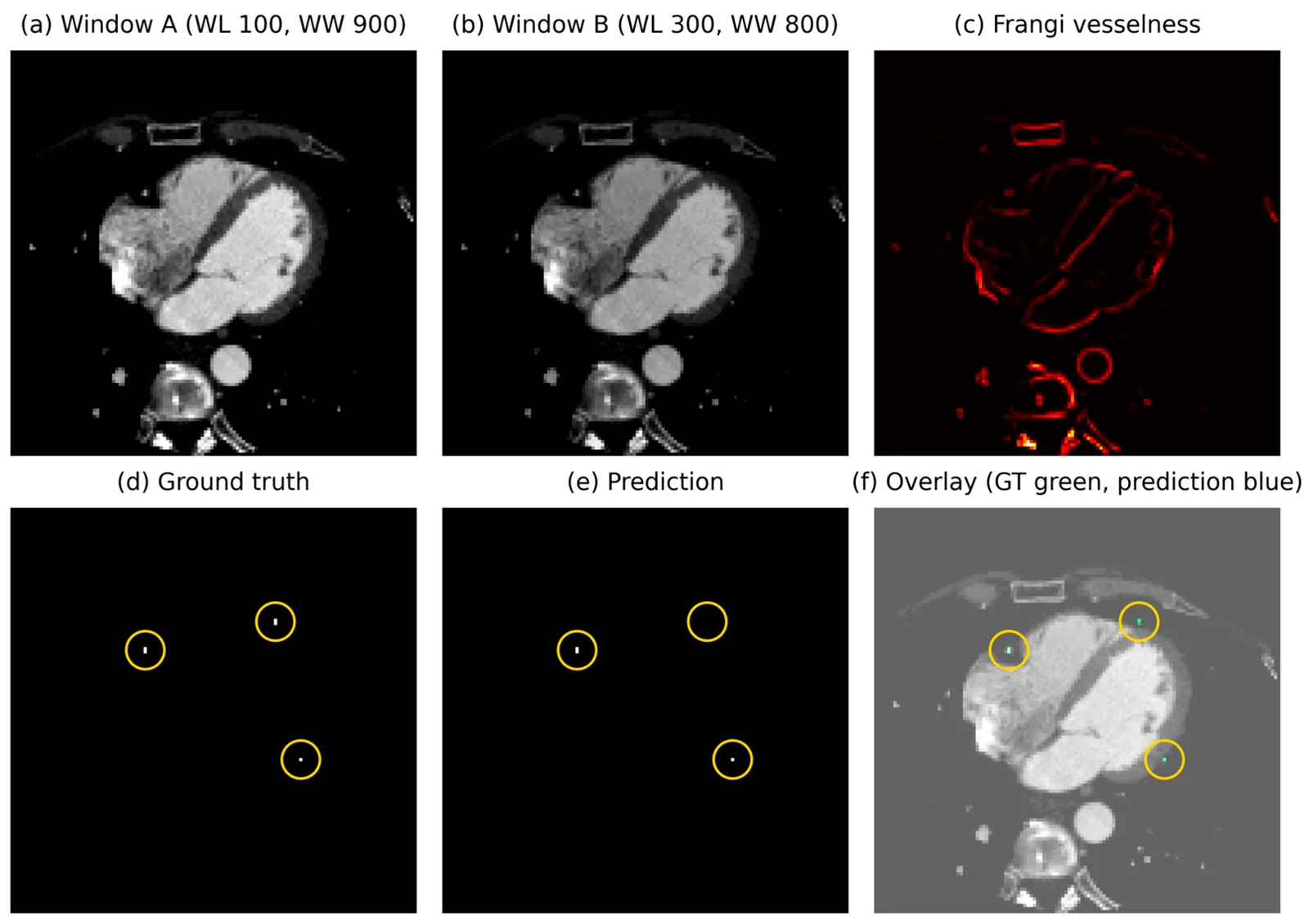
Representative test sample showing the CT image under both selected HU windows, the Frangi vesselness map, ground-truth mask, model prediction, and segmentation overlay (ground truth in green, prediction in blue and overlapping pixels in cyan). Yellow circles mark the three reference vessel cross-sections in this slice; two of them are detected by the model.

**Figure 8:**
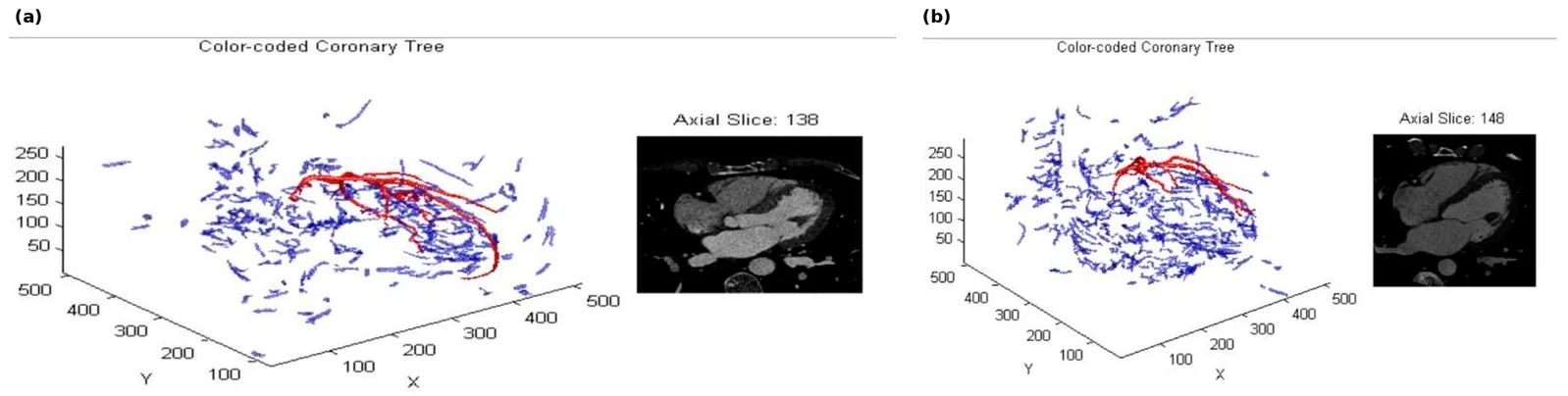
Three-dimensional reconstruction of the coronary vessel segmentation across a range of slices, with the main traced vessel path shown in red and other predicted structures shown in blue. Produced with the model trained under the slice-level split; qualitative visualization only. (**a**) Reconstruction displayed together with axial slice 138; (**b**) reconstruction displayed together with axial slice 148.

Figure 7 presents an example from the test set and illustrates the output of the proposed dual-window fusion framework. The CT images generated using the two selected HU windows show the same cardiac region with differences in contrast representation, allowing complementary intensity information to be retained. Small coronary vessel regions are visible within the cardiac anatomy, although their appearance varies depending on the selected window. The corresponding Frangi vesselness map enhances tubular and vessel-like structures within the image. However, this response is not limited to the coronary arteries and may also highlight other anatomical structures with similar tubular characteristics. Therefore, the Frangi representation was not used as a standalone segmentation output. Instead, it was provided as an additional input feature and combined with the original CT information and Gaussian-smoothed representation. The final segmentation was then obtained by jointly learning from these complementary features across both HU window branches. The prediction reproduces most of the reference vessel cross-sections, with boundary-level discrepancies concentrated at the smallest and most distal structures, consistent with the boundary-accuracy characteristics reflected in the HD95 values reported in Section 4.2. Figure 8 shows a three-dimensional view of the coronary tree obtained by stacking the 2D predictions of the model trained under the slice-level split; the main traced vessel path is shown in red and other predicted structures in blue. The main vessel path remains continuous across consecutive slices, although isolated predicted structures are also visible. This view is qualitative.

### 4.7. Explainability: Grad-CAM

**Figure 9:**
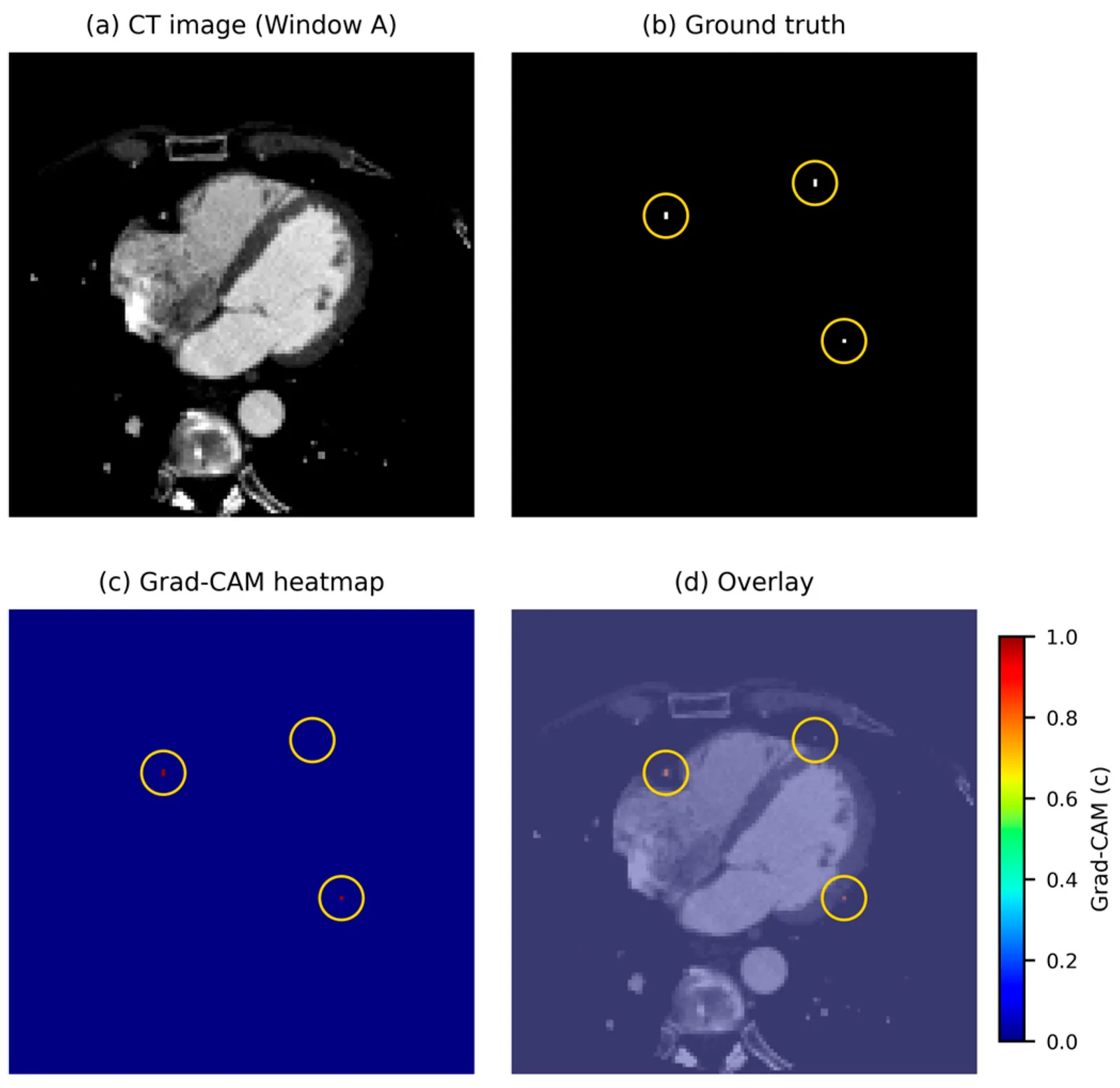
Grad-CAM activation heatmap and overlay for a test sample: (**a**) CT image, (**b**) ground truth, (**c**) Grad-CAM heatmap, (**d**) overlay. Yellow circles mark the reference vessel cross-sections.

The Grad-CAM result for a test sample is shown in Figure 9. The activations were sparse and concentrated at a few locations near the coronary vessel cross-sections, while most of the image showed little activation. Because the activation map is very sparse, Grad-CAM gives only limited insight into the model’s decisions here, and it is used as a qualitative illustration.

### 4.8. MC-Dropout Uncertainty and Calibration

**Figure 10:**
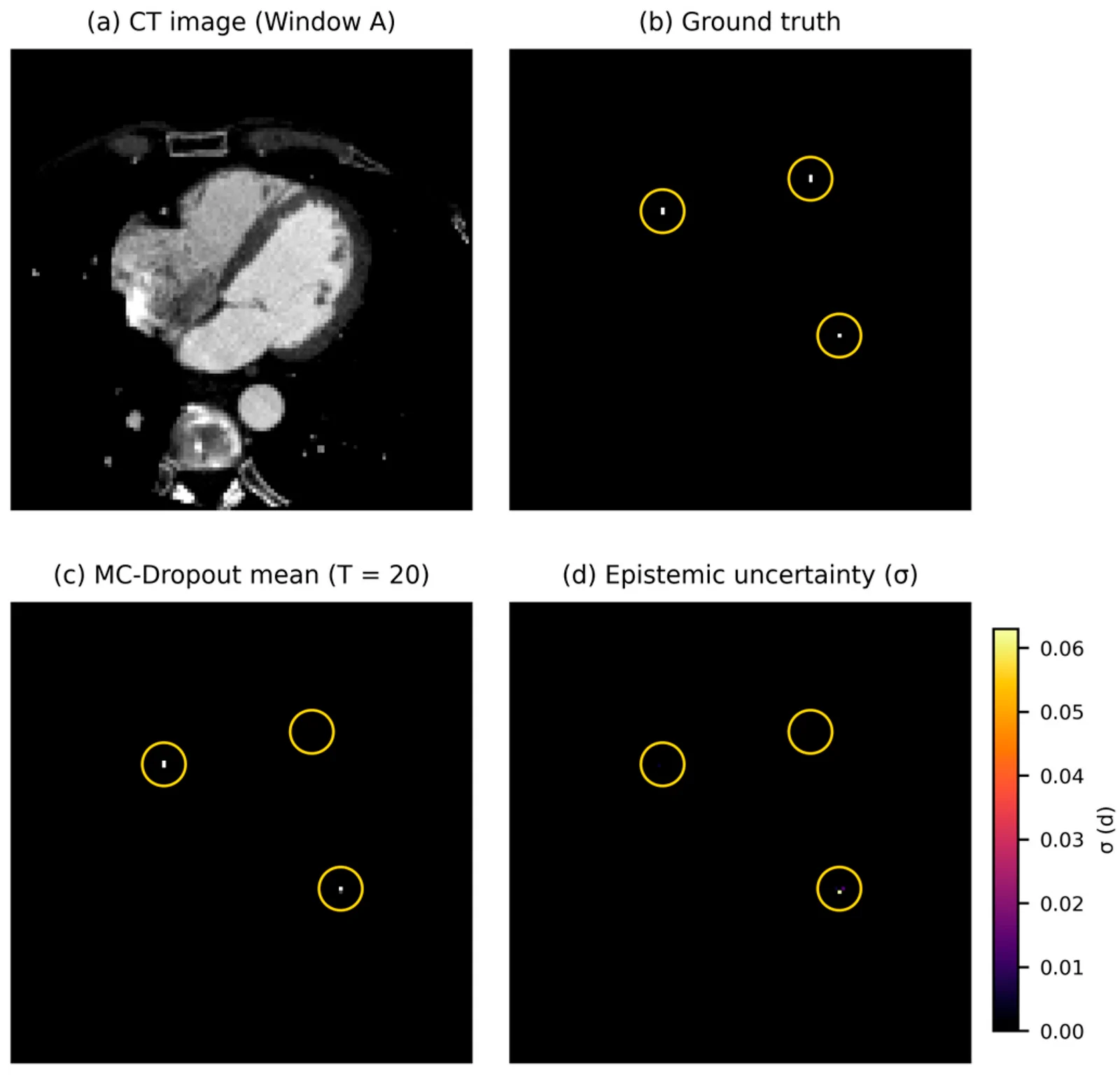
MC-Dropout mean prediction and epistemic uncertainty map for a test sample (*T* = 20): (**a**) CT image, (**b**) ground truth, (**c**) MC-Dropout mean prediction, (**d**) epistemic uncertainty (standard deviation σ). Yellow circles mark the reference vessel cross-sections.

**Figure 11:**
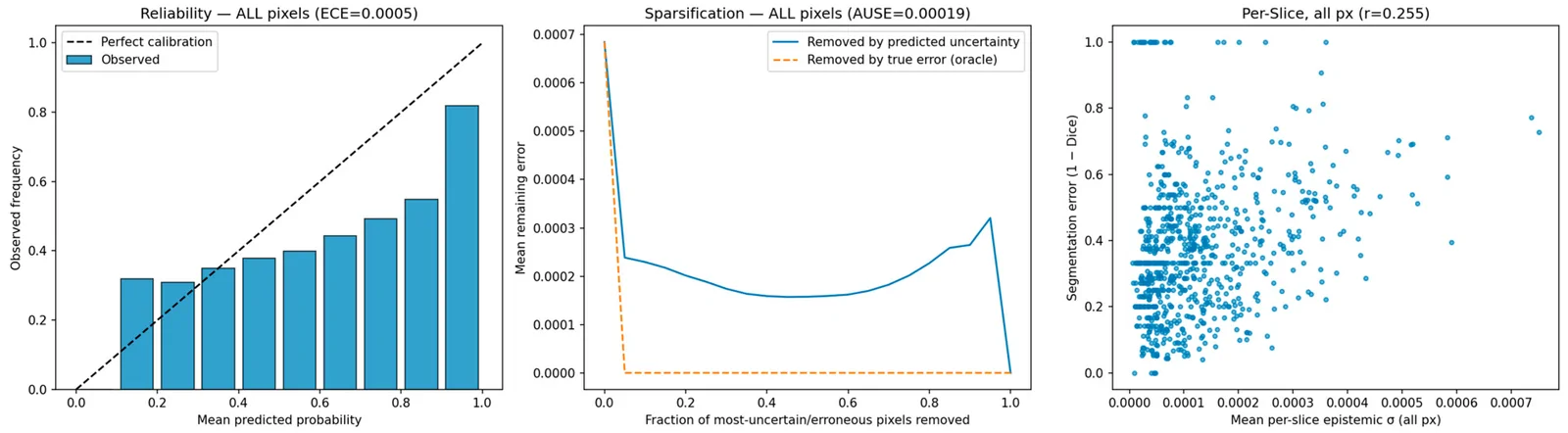
Uncertainty calibration on the 777 test slices: reliability diagram (**left**), sparsification curve (**middle**) and per-slice mean uncertainty versus segmentation error (**right**).

Figure 10 shows the MC-Dropout mean prediction and the epistemic uncertainty map (*T* = 20) for a test sample. The uncertainty was low in the background and higher at the boundaries of the predicted vessels and at small distal cross-sections, where the vessels are small and the local contrast is low. Over all pixels of the 777 test slices, the expected calibration error was 0.0005 and the AUSE was 0.00019 (Figure 11); both values are dominated by the large background. At slice level, the mean epistemic uncertainty correlated only modestly with the segmentation error (Pearson *r* = 0.255). A reliability analysis restricted to the reference vessel pixels is not informative, because all of these pixels have the positive label. The uncertainty maps therefore show regions of less consistent prediction, but they are not a validated error detector.

### 4.9. Computational Efficiency

As shown in Table 11, the dual encoder increases the number of parameters by a factor of 1.68 and the FLOPs by a factor of 1.36 compared with the single-encoder models, but inference for a complete patient still takes about 0.3 s. The total time of about 20 s per patient is dominated by the CPU pre-processing (two windows, Frangi and Gaussian channels).

### 4.10. Robustness to Contrast Variation and Calcification

Table 12 shows the effect of synthetic HU shifts. These analyses were computed in a separate script that re-reads the test slices from raw HU and applies the trained main model; its baseline without shift (Dice 0.679) differs slightly from Table 5, so all values in Table 12 and the calcification subgroup are given relative to this baseline. When the shift was applied only to enhanced voxels, simulating stronger or weaker iodine enhancement, Dice changed by at most 0.040 for shifts up to ±50 HU. A global offset of all voxels, which corresponds to a calibration error, reduced the performance substantially, showing that fixed-window methods depend on correct HU calibration. For the calcification analysis, vessel slices were labeled as calcified if at least three pixels above 600 HU were within five pixels of the reference lumen (per-patient mean lumen attenuation over all 16 patients: 174–457 HU); 800 HU was used as a sensitivity check. Only 17 of the 777 test slices (all of which contain vessel), from two patients, met the 600 HU criterion and none met the 800 HU criterion. The Dice in these slices (0.812) was higher than in the non-calcified slices (0.676), but the subgroup is too small and too concentrated in two patients for a conclusion.

## 5. Comparison with Existing Literature

Many published coronary segmentation methods use different datasets, modalities (including X-ray angiography) and protocols [50,51,52,53,54], and HD95 values in different units. They are therefore not compared numerically here. The directly comparable evidence of this study is the comparison with single-window and two-channel models trained on the same patients and folds (Table 6 and Table 7). Table 13 summarizes published work on the Rotterdam data for context. The volumes used in this study originate from the training set of the Rotterdam lumen segmentation framework [43], while the MICCAI 2008 centerline challenge [42] uses centerline overlap measures (OV, OF, OT) instead of Dice.

Among these studies, Wolterink et al. [55] report the Dice coefficient and Jawaid et al. [57] the Jaccard index (IoU). Wolterink et al. [55] obtained a higher Dice (0.75 and 0.73) than our held-out Dice of 0.691, but their network starts from a vessel centerline and the lumen is evaluated segment by segment around the reference centerline, so false positives far from the vessel do not affect their score. Jawaid et al. [57] evaluated a hybrid level-set model on the same 18 Rotterdam training volumes and obtained an IoU of 0.716 for healthy and 0.656 for diseased segments, compared with our IoU of 0.580; their method is initialized from coronary seed points, and the IoU is computed per segment on 2D cross-sections orthogonal to the reference centerline, so only the lumen region of each cross-section is evaluated. Han et al. [56] require a seed point at each coronary ostium and report centerline overlap rather than area overlap. In contrast, DWFAU-Net performs direct segmentation of the full CT slice: it receives only the image and uses no region of interest (ROI) crop around the heart or the vessels, no centerline, no seed point and no manual interaction. Its Dice and IoU are therefore computed over whole slices, in which the coronary lumen occupies a very small fraction of the pixels, so every false-positive pixel anywhere in the slice lowers the score. This is likely a main reason why our Dice values are lower than those of the centerline-based methods, whose evaluation is restricted to the region around the vessel. The main gap addressed by this work is therefore fully automatic, image-only lumen segmentation; such a segmentation could also provide the centerline that the centerline-based methods require. In addition, at 128 × 128 pixels the coronary vessels are only a few pixels wide, so small boundary errors change the Dice considerably. The results should not be viewed as a definitive ranking of the compared methods because of differences in the datasets used, in the scanner characteristics, in the preprocessing procedures, in the training strategies, and in the evaluation protocols. A reliable ranking would require all methods to be evaluated independently under the same experimental conditions.

## 6. Discussion

A rigorous validation protocol is critical for translating deep learning models into clinical cardiac workflows. In this study, the proposed framework was evaluated with two splitting strategies on the Rotterdam dataset. The slice-level protocol yielded a Dice of 0.870, whereas the strict patient-level protocol gave 0.691 on three held-out patients and a volumetric Dice of 0.565 ± 0.111 in five-fold cross-validation. Although this drop appears steep, the patient-level values represent the genuine generalization of the model to entirely unseen patients, and we recommend that studies on small CCTA cohorts report patient-level results.

The main strengths of this study are its evaluation design and the transparency of its comparisons: all model-selection decisions were made on validation patients, the proposed model was compared with single-window and concatenation baselines trained with an identical recipe, per-patient paired statistics with multiple-comparison correction and bootstrap confidence intervals were reported, the variation between repeated trainings was measured, and all results were obtained on whole slices without a region of interest, centerline or seed points. These choices make the reported values lower than those of slice-level or ROI-based evaluations, but they are reproducible and representative of fully automatic use on unseen patients.

Under identical training, the dual-window fusion model performed comparably to the single-window and two-channel concatenation models, with the lowest mean HD95 but no statistically significant difference with 16 patients. It achieved the highest slice Dice of the three models in each of folds 1–4, and its slightly lower mean overlap over all 16 patients was caused entirely by a marked drop in fold 5 (slice Dice 0.257 versus 0.405 for the single-window model), which was reproduced with a second training seed. We therefore present dual-window fusion as a competitive design that achieved the best boundary accuracy, whose overlap advantage in folds 1–4 should be confirmed on larger cohorts, at a moderate computational cost (1.68× the parameters of the single-encoder model and about 0.3 s of inference per patient). The two-channel baseline received only the two windowed CT images, without the Frangi and Gaussian channels, so its comparison with DWFAU-Net reflects both the second encoder and the auxiliary channels.

The information in the Frangi channel is related to the tubular structures and can therefore be used to identify vessel-like regions. However, vesselness responses are not restricted to the coronary arteries and can also be seen in other anatomical structures. It is therefore possible that the information from the original CT scan, as well as the Gaussian-smoothed context, could help the network to differentiate between vessel-related structures and other structures in the vicinity. The patient-level ablation with five seeds supports this interpretation: without the Frangi channel, the mean Dice decreased from 0.697 to 0.677 and the volumetric Dice from 0.712 to 0.684, the full model was better in four of the five seeds and in all three test patients, and its results varied less between seeds (Table 10). This gain was consistent across seeds and test patients; it did not reach statistical significance, which is not attainable at the 0.05 level with five paired trainings (smallest two-sided Wilcoxon *p* = 0.0625), and it indicates that the vesselness prior helps the network on unseen patients. Under the slice-level protocol, in contrast, the channel configurations did not differ beyond the variation of repeated trainings (Table 9), probably because the leakage between training and test slices makes all variants perform similarly. At a weight of 0.5, the clDice term gave the best Dice and HD95 of the tested weights in the slice-level ablation and preserved overlap at the patient level while slightly increasing skeleton precision, although these differences were within the variation of repeated trainings, whereas a higher weight (1.0) clearly degraded the slice-level results, so the clDice weight is an important hyperparameter. In the slice-level ablation, the difference between the Attention U-Net and the plain U-Net (Dice 0.881 versus 0.866) was within the variation of repeated trainings; the individual benefit of clDice at *w* = 0.5 and of the attention gates therefore requires confirmation on a larger cohort.

The HD95 values should also be taken into account. The mean HD95 of 22.20 pixels on the held-out patients had a large standard deviation, indicating that boundary errors varied strongly between slices. This variation is expected for coronary CT images, in which branches and bifurcation regions, as well as partially visible elongated structures, are more challenging to segment. These areas are illustrated by the uncertainty maps in Figure 10. The increased MC-Dropout uncertainty was primarily found in areas close to vessels and in small distal structures. The small vessel diameter, partial-volume effects and low local contrast make these areas difficult. The quantitative calibration analysis showed only a modest correlation between uncertainty and segmentation error (*r* = 0.255), so the uncertainty maps should be interpreted qualitatively. The model was robust to changes in contrast enhancement of up to ±50 HU but sensitive to global HU offsets.

### 6.1. Practical Implications

From an implementation perspective, the proposed framework may have potential for future use in automated coronary artery analysis. The segmentation masks could serve as an initial delineation for subsequent expert review, and the uncertainty maps may help highlight regions where the model produces less consistent predictions. However, the model was evaluated within the experimental setting of this study, on 16 patients from a single public dataset, and its performance under the wider range of image quality and acquisition conditions encountered in routine clinical practice remains to be validated using larger real-world clinical datasets; the method is therefore not yet suitable for clinical use without further validation.

### 6.2. Limitations

The proposed framework has several limitations. First, the framework was only tested with the public Rotterdam dataset, with 16 annotated volumes, and no external dataset was evaluated. Between-patient variability was large (per-patient VolDice of DWFAU-Net 0.380–0.728), and the statistical power to detect small differences between models is limited; evaluation on external cohorts such as ASOCA [58] or ImageCAS [59] is needed. Second, HD95 is reported in pixels because the pixel spacing was not available in the DICOM files, so it cannot be compared with millimeter values reported elsewhere. Third, the network is 2D and operates at 128 × 128 pixels, which limits the depiction of thin distal branches. Fourth, performance degraded under global HU offsets, and the held-out patients contained very little calcification, so the effect of calcium blooming could not be assessed. Fifth, the cross-validation used a single training seed per fold, and per-patient results varied by up to 0.163 VolDice between seeds in the repeated fold; with only three or four test patients per fold, fold-level estimates are inherently variable [60], which is consistent with the lower result in fold 5. Sixth, the HU windows were ranked once on the validation patients of the main split and kept fixed in all cross-validation folds, so two of the cross-validation test patients had contributed to window ranking. Because the same windows were used by all compared models (Window A by the single-window model and both windows by the concatenation model), this affects the three models equally, and the fold containing patient 03 (fold 5) was the weakest fold of DWFAU-Net, so this overlap did not favor the proposed model. Seventh, most component ablations were performed under the slice-level protocol; the patient-level ablation covered only the clDice term and the Frangi channel, with five seeds on three test patients, so the gain from the Frangi channel, although consistent, could not reach statistical significance (with five paired seeds, the smallest attainable two-sided Wilcoxon *p*-value is 0.0625). In addition, only the two best-ranked windows were fused, as required by the dual-encoder design, and other window combinations were not tested. Finally, the comparison was limited to 2D Attention U-Net variants trained with an identical recipe; self-configuring 3D frameworks such as nnU-Net [61] were not included and remain a reference point for future work.

## 7. Conclusions

In this study, a coronary artery segmentation framework was developed that fuses two HU windows within an Attention U-Net architecture. Each HU window was represented by three input channels: the normalized CT image, the Frangi vesselness response and a Gaussian-smoothed image. The network used attention-gated skip connections and a composite BCE + Soft-Dice + clDice loss, and its behavior was examined with Grad-CAM and MC-Dropout uncertainty, whose calibration was quantified. Under a strict patient-level protocol, the model segmented the coronary lumen of unseen patients with a Dice of 0.691 (IoU 0.580), a mean Dice of 0.697 ± 0.012 over five further trainings, and a volumetric Dice of 0.565 ± 0.111 in five-fold cross-validation, without requiring a region of interest, a centerline or seed points. The model was robust to contrast-enhancement shifts of up to ±50 HU and processed a complete patient in about 0.3 s. It performed comparably to single-window and two-channel concatenation models trained under identical conditions, achieving the lowest mean HD95 and the highest slice Dice in four of the five folds, although the differences were not statistically significant with 16 patients; dual-window fusion is therefore a competitive design with the best boundary accuracy. Among the design elements, the Frangi vesselness channel gave the most consistent gain, raising the mean volumetric Dice from 0.684 to 0.712, with a higher value in all three test patients, and reducing the variation between trainings, and a clDice weight of 0.5 gave the best Dice and HD95 of the tested weights while preserving overlap at the patient level, showing that the topology-aware term can be included without loss of accuracy when its weight is tuned (a weight of 1.0 degraded the results); the contribution of the attention gates could not be separated from run-to-run variation in the slice-level ablation. The same architecture reached a Dice of 0.870 under a slice-level split, which shows that the evaluation protocol affects the reported accuracy more than the architectural choices examined and that patient-level evaluation is essential on small CCTA datasets. These results provide a realistic, statistically tested baseline and practical recommendations for future work. However, the evaluation was limited to 16 volumes from a single public dataset, and the performance of the model on data from other institutions, scanners and acquisition protocols has not yet been confirmed. Further validation on larger external datasets is required before the method can be considered for clinical use.

### Future Work

Several aspects of the proposed framework can be explored further. The benefit of the Frangi channel should be confirmed on larger and external datasets, the contributions of the clDice term, the attention gates and dual-window fusion should be re-examined on more patients, and the HU windows should be selected inside each cross-validation fold. The present framework fuses two windows selected from literature-guided candidates; a larger set of window combinations or a learnable window-selection mechanism could provide an intensity range tailored to segmentation instead of pre-set values, and intensity-shift augmentation may reduce the sensitivity to HU calibration. Extending the model to 2.5D or 3D input and to a higher input resolution is also expected to improve vessel continuity. Finally, external validation on data from different institutions, scanner vendors and acquisition protocols, for example ASOCA [58] and ImageCAS [59], is needed to establish the robustness of the framework. Beyond the proposed framework, the present results suggest several practices for future studies on small CCTA datasets: data should be split by patient rather than by slice, all model-selection decisions should be made on validation patients, vessel-oriented input features such as the Frangi vesselness map should be considered as a low-cost addition, and boundary, centerline and robustness measures should be reported in addition to the Dice coefficient.

## Figures and Tables

**Table 1:** Composition of multi-vendor Rotterdam Dataset.

Attribute	Rotterdam–Siemens	Rotterdam–Toshiba	Rotterdam–Philips
Vendor	Siemens	Toshiba	Philips
Volume count	6	6	6
Institution	Erasmus Univ. (NL)	Leiden Univ. (NL)	Utrecht Univ. (NL)
CT scanner	Somatom Definition	Aquilion One 320	Brilliance 64
Slices × rotation	32 × 2	320 × 1	64 × 1
ECG gating	Retrospective	Retrospective	Retrospective
Reconstruction kernel	b26f	b26f	b26f
Contrast medium	Ultravist 370	Ultravist 370	Ultravist 370

**Table 2:** HU window configurations evaluated in this study; the two best-ranked windows were fused.

Window Label	WL	WW	Source/Clinical Rationale
Conventional coronary window (Window A)	100	900	Vendor-default reference window for conventional images [12]
Standard CCTA review (Window B)	300	800	Initial window of the SCCT guidelines [11,44]
Fixed plaque-analysis window	220	740	Validated against IVUS for plaque volume analysis [45]
Narrow lumen window	200	600	Added empirically; no published source

**Table 3:** Experimental Setup.

Parameters	Description
Language	Python 3.12
GPU Memory	16 GB
Computing Environment	Google Colab
Hardware	NVIDIA T4 GPU (NVIDIA Corporation, Santa Clara, CA, USA)
Training Optimization	Mixed-precision training used to reduce activation memory and utilize T4 Tensor Cores
Random seed	42
Model Implementation	TensorFlow 2.19/Keras
Vessel Enhancement	Frangi vesselness filtering using scikit-image
DICOM processing	Pydicom used for input/output operations
Hausdorff distance	Computed using scipy.spatial.cKDTree
Epochs	Maximum 70
Optimizer	Adam
Batch Size	8
Dropout rate	0.2 (after the bottleneck and the first two decoder blocks)
Learning Rate	1 × 10^−4^
Learning rate scheduler	ReduceLROnPlateau (reduction factor 0.8, patience 8)
Early stopping	15 epochs; restore best weights (monitored on validation patients)
Augmentation	Horizontal flip, rotation (up to ±21.6°), translation (up to ±8%); vessel slices oversampled × 3
Decision threshold	Selected on validation patients (0.25 for the final model)

**Table 4:** HU window grid search results (5-epoch proxy training, evaluated on validation patients).

Window	WL	WW	Best Threshold	Validation IoU
Conventional (Window A)	100	900	0.45	0.308
Standard CCTA Review (Window B)	300	800	0.30	0.264
Fixed plaque-analysis window	220	740	0.30	0.254
Narrow lumen window	200	600	0.30	0.168

**Table 5:** Final performance of the Dual-Window Fusion Attention U-Net under the two data-splitting protocols (mean ± standard deviation).

Metric	Slice-Level Split	Patient-Level Split
IoU	0.783 ± 0.175	0.580 ± 0.187
Dice coefficient	0.870 ± 0.140	0.691 ± 0.172
Sensitivity (Recall)	0.859 ± 0.168	0.697 ± 0.222
Specificity	0.9999 ± 0.0001	0.9997 ± 0.0004
HD95 (px)	2.70 ± 7.51	22.20 ± 22.79
Skeleton recall	—	0.697 ± 0.252
Skeleton precision	—	0.702 ± 0.273
Overlap until first error	—	0.370 ± 0.414

Slice-level split: slices of the same patient could be in both training and test sets (data leakage); reported for comparison only. Patient-level split: three unseen test patients (777 slices); all model selection on validation patients. Centerline metrics were not computed for the slice-level split. Specificity is given to four decimals because all values are close to 1.

**Table 6:** Five-fold patient-level cross-validation (mean ± SD over 16 patients). Specificity was 0.9997 for all models.

Model	Dice	VolDice	IoU	Sensitivity	HD95 (px)
Single-window Attention U-Net	0.503 ± 0.143	0.583 ± 0.096	0.383 ± 0.125	0.512 ± 0.165	31.96 ± 11.58
2-channel concatenation (single encoder)	0.482 ± 0.157	0.559 ± 0.122	0.364 ± 0.138	0.499 ± 0.160	31.21 ± 10.85
DWFAU-Net (dual encoder)	0.495 ± 0.169	0.565 ± 0.111	0.376 ± 0.144	0.504 ± 0.194	30.55 ± 10.94

**Table 7:** Paired comparison (per-patient values, *n* = 16; two-sided Wilcoxon signed-rank test; Holm correction per metric over the two comparisons).

DWFAU-Net vs.	Metric	Mean Difference	95% CI	*p*	*p* (Holm)
Single window	VolDice	−0.018	[−0.054, 0.011]	0.90	1.00
Single window	Dice	−0.008	[−0.052, 0.029]	0.82	1.00
Single window	HD95 (px)	−1.41	[−3.72, 0.88]	0.23	0.46
2-ch concatenation	VolDice	+0.006	[−0.044, 0.063]	0.94	1.00
2-ch concatenation	Dice	+0.013	[−0.045, 0.075]	0.78	1.00
2-ch concatenation	HD95 (px)	−0.66	[−4.02, 2.10]	0.98	0.98

**Table 8:** Per-fold results (slice Dice/VolDice).

Fold	Test Patients	DWFAU-Net	Single Window	2-ch Concat
1	00, 01, 05, 14	0.656/0.653	0.638/0.658	0.625/0.654
2	08, 11, 13	0.455/0.548	0.393/0.530	0.429/0.523
3	02, 09, 15	0.576/0.613	0.558/0.591	0.532/0.555
4	04, 07, 10	0.479/0.542	0.475/0.542	0.387/0.492
5	03, 06, 12	0.257/0.439	0.405/0.568	0.391/0.537

**Table 9:** Ablation results under the slice-level protocol (single-window Attention U-Net unless stated; at most 70 epochs with early stopping; comparison between variants only).

Configuration	IoU	Dice	Sensitivity	HD95 (px)
Input channels				
A1: CT only	0.789 ± 0.170	0.870 ± 0.135	0.881	2.23
A2: CT + Gaussian (no Frangi)	0.788 ± 0.175	0.868 ± 0.144	0.862	1.80
A3: CT + Frangi (no Gaussian)	0.786 ± 0.168	0.868 ± 0.132	0.867	2.39
A4: CT + Frangi + Gaussian †	0.794 ± 0.170	0.873 ± 0.137	0.874	2.09
Architecture				
B1: Plain U-Net	0.785 ± 0.177	0.866 ± 0.142	0.861	2.86
B2: Attention U-Net †	0.805 ± 0.160	0.881 ± 0.126	0.882	1.77
Base loss function				
C1: BCE only	0.797 ± 0.161	0.877 ± 0.129	0.877	1.79
C2: Focal + Dice	0.792 ± 0.158	0.874 ± 0.130	0.870	1.51
C3: BCE + Soft-Dice †	0.798 ± 0.163	0.878 ± 0.137	0.869	1.57
Frangi scale range				
D1: σ = 1–2	0.779 ± 0.173	0.863 ± 0.137	0.861	2.15
D2: σ = 1–4 †	0.796 ± 0.165	0.875 ± 0.129	0.876	1.69
D3: σ = 2–6	0.793 ± 0.170	0.872 ± 0.137	0.883	1.69
D4: σ = 1–6	0.782 ± 0.180	0.863 ± 0.149	0.853	2.36
clDice weight				
F1: BCE + Soft-Dice (no clDice) †	0.786 ± 0.166	0.869 ± 0.129	0.886	1.85
F2: + clDice (*w* = 0.25)	0.778 ± 0.166	0.863 ± 0.133	0.865	2.18
F3: + clDice (*w* = 0.5)	0.792 ± 0.172	0.871 ± 0.139	0.878	1.69
F4: + clDice (*w* = 1.0)	0.777 ± 0.178	0.860 ± 0.147	0.852	2.93
Window fusion				
G0: 2-channel concatenation, single encoder	0.777 ± 0.177	0.861 ± 0.143	0.855	2.42
G1: Single window	0.782 ± 0.167	0.866 ± 0.128	0.863	2.44
G2: 6-channel concatenation, single encoder	0.782 ± 0.172	0.865 ± 0.138	0.862	2.46
G3: Dual-window fusion (DWFAU-Net)	0.783 ± 0.175	0.870 ± 0.140	0.859	2.70

† Identical configurations; together with the Standard-CCTA window run (Dice 0.868), six trainings of the same configuration gave Dice 0.868–0.881. HD95 in pixels (no pixel spacing in the files). G3 is the slice-level run reported in Table 5 (fixed threshold 0.5).

**Table 10:** Patient-level ablation of the clDice term and the Frangi channel (three held-out test patients, 777 slices; mean ± SD over five training seeds).

Variant (DWFAU-Net)	Dice	VolDice	IoU	HD95 (px)	Skel. Recall	Skel. Precision	OUFE
Main model (Section 4.2)	0.691	—	0.580	22.20	0.697	0.702	0.370
P0: full model	0.697 ± 0.012	0.712 ± 0.009	0.561 ± 0.012	22.11 ± 2.93	0.706 ± 0.038	0.705 ± 0.053	0.440 ± 0.053
P1: without clDice	0.696 ± 0.018	0.711 ± 0.017	0.560 ± 0.022	21.02 ± 1.76	0.714 ± 0.018	0.690 ± 0.045	0.435 ± 0.008
P2: without Frangi channel	0.677 ± 0.028	0.684 ± 0.026	0.539 ± 0.027	22.26 ± 1.07	0.708 ± 0.039	0.674 ± 0.026	0.434 ± 0.050

P0–P2 were trained with identical settings and seeds 42, 7, 1, 2 and 3; the main model has the same configuration as P0 and was trained in a separate run (results differ between runs with the same seed because of non-deterministic GPU operations). OUFE: overlap until first error. VolDice was not computed in the main run.

**Table 11:** Computational cost on an NVIDIA T4 GPU (one patient, 222 slices). Pre-processing took 19.7 s per patient on the CPU. Latency/slice was measured with one slice per call (batch size 1) and includes the per-call overhead; inference/patient was measured with all slices of the patient processed in batches.

Model	Params (M)	GFLOPs/Slice	Latency/Slice, bs 1 (ms)	Inference/Patient (s)	Peak GPU (MB)
DWFAU-Net (dual encoder)	3.34	7.45	143.9	0.31	504
Single-window Attention U-Net	1.99	5.48	101.1	0.25	439
2-channel concatenation	1.98	5.47	100.3	0.23	411

**Table 12:** Change in Dice on the held-out patients under synthetic HU shifts.

Shift Applied to	−50 HU	−25 HU	+25 HU	+50 HU
Enhanced voxels only (>150 HU)	+0.010	+0.010	−0.017	−0.040
All voxels (global offset)	−0.184	−0.034	−0.196	−0.373

**Table 13:** Published work on the Rotterdam coronary data (context only, not a ranking). Values as reported in the original publications; OV, OF and OT converted from percentages to decimals.

Study	Data/Split	Method and Required Input	Dice/IoU (As Reported)	Evaluation Region	Key Difference from DWFAU-Net
Wolterink et al. [55]	Rotterdam lumen framework; 78 vessel segments	Graph convolutional network; needs a centerline (mesh anchored to centerline points)	Dice 0.75 (healthy)/0.73 (diseased); IoU not reported	Segment by segment around the centerline	Centerline must be extracted first, and background far from the vessel is not evaluated
Han et al. [56]	Rotterdam CAT08; 32 scans (8 training + 24 test)	Bayesian tracking; needs one seed point at each coronary ostium	Dice and IoU not reported (OV 0.836, OF 0.653, OT 0.882)	Centerline overlap	Measures centerline tracking, not lumen area overlap
Jawaid et al. [57]	Rotterdam lumen framework; 18 training volumes	Hybrid local/global level-set model (not deep learning); needs coronary seed points for initialization	IoU 0.716 (healthy)/0.656 (diseased); Dice not reported	Per segment, on 2D cross-sections orthogonal to the reference centerline	Evaluated only on vessel cross-sections; needs seed points
DWFAU-Net (this work)	16 volumes; patient-level split and 5-fold CV	2D dual-window fusion Attention U-Net; image only, no centerline or seed point	Dice 0.691/IoU 0.580 (held-out); VolDice 0.565 (CV)	Whole slice (no ROI) at 128 × 128 pixels	Direct full-slice segmentation; no ROI crop, centerline or seed; every false positive in the slice is counted

## Data Availability

The Rotterdam data are publicly available from the framework organizers [43]. The data that support the findings of this study are available from the corresponding author upon reasonable request.
